# Role of NF-E2 related factor 2 (Nrf2) on chemotherapy resistance in acute myeloid leukemia (AML) and the effect of pharmacological inhibition of Nrf2

**DOI:** 10.1371/journal.pone.0177227

**Published:** 2017-05-15

**Authors:** Sreeja Karathedath, Bharathi M. Rajamani, Syed Mohammed Musheer Aalam, Ajay Abraham, Savitha Varatharajan, Partha Krishnamurthy, Vikram Mathews, Shaji Ramachandran Velayudhan, Poonkuzhali Balasubramanian

**Affiliations:** 1 Department of Haematology, Christian Medical College, Vellore, India; 2 Centre for Stem Cell Research, Christian Medical College, Vellore, India; 3 Department of Pharmacology, Toxicology and Therapeutics, Kansas University Medical Centre, Kansas City, Kansas, United States of America; North Carolina State University, UNITED STATES

## Abstract

Cytarabine (Ara-C) and Daunorubicin (Dnr) forms the backbone of acute myeloid leukemia (AML) therapy. Drug resistance and toxic side effects pose a major threat to treatment success and hence alternate less toxic therapies are warranted. NF-E2 related factor-2 (Nrf2), a master regulator of antioxidant response is implicated in chemoresistance in solid tumors. However, little is known about the role of Nrf2 in AML chemoresistance and the effect of pharmacological inhibitor brusatol in modulating this resistance. Primary AML samples with high *ex-vivo* IC50 to Ara-C, ATO, Dnr had significantly high *NRF2* RNA expression. Gene-specific knockdown of *NRF2* improved sensitivity to these drugs in resistant AML cell lines by decreasing the expression of downstream antioxidant targets of Nrf2 by compromising the cell’s ability to scavenge the ROS. Treatment with brusatol, a pharmacological inhibitor of Nrf2, improved sensitivity to Ara-C, ATO, and Dnr and reduced colony formation capacity. AML cell lines stably overexpressing *NRF2* showed increased resistance to ATO, Dnr and Ara-C and increased expression of downstream targets. This study demonstrates that Nrf2 could be an ideal druggable target in AML, more so to the drugs that function through ROS, suggesting the possibility of using Nrf2 inhibitors in combination with chemotherapeutic agents to modulate drug resistance in AML.

## Introduction

Acute myeloid leukemia (AML) is a clinically and biologically heterogeneous malignancy characterized by increased proliferation and defective maturation of the cells of the myeloid lineage. The frontline treatment for AML (except for AML-M3) involves the combination of Cytarabine (Ara-C) and Daunorubicin (Dnr). Although ~70–85% of the patients achieve initial induction remission with this treatment, the 5-year overall survival is only 30–40%, with drug resistance being the major cause of the treatment failure [1]. Also, toxic effects to these drugs limit the success of this therapy in AML patients with pre-existing comorbidities [2]. Moreover, treatment-related mortality is reported in >30% of older AML patients with poor performance factor receiving standard induction chemotherapy [3] thereby warranting alternative less toxic therapies in this group of patients.

Some of the major factors contributing to drug resistance in AML include the persistence of leukemic stem cells [4], altered expression of drug influx and efflux transporters [5], altered cell cycle checkpoints [6] and increased antioxidant defense systems[7]. Novel therapeutic strategies against these targets such as inhibitors of cell cycle proteins [8], pro-apoptotic proteins [9] and drug transporters [10] have been studied to overcome drug resistance. Targeting the antioxidant defense pathways are also shown to be an effective strategy for eliminating cancer cells [11]. Constitutive overexpression of NF-E2 related factor 2 (*NFE2L2 or NRF2*), the master regulator of antioxidant pathway genes involved in scavenging and detoxification of reactive oxygen species (ROS) results in chemoresistance as reported in several solid tumors ([12] [13] [14]). Overexpression of Nrf2 results either from the mutations [15] or epigenetic modifications [16] in its binding partner KEAP1, transcriptional activation of *NRF2* by oncogenic RAS or c-MYC [17] or due to phosphorylation at the serine, threonine or tyrosine residues which prevents their ubiquitination [18].

There are limited studies suggesting the role of Nrf2 in mediating chemoresistance in hematological malignancies. AML cells have been shown to express high nuclear levels of Nrf2, and its knockdown improved the chemosensitivity to Ara-C and Dnr [19]. Studies in NCI-60 tumor cell lines panel suggested that Nrf2 mediated oxidative stress response pathways are enriched in arsenic trioxide (ATO) resistant tumor cell lines [20]. The reduced activity of ATO on non-M3 AML cells could hence be attributed to the increased expression of Nrf2.

Pharmacological inhibition of Nrf2 has been shown to improve the sensitivity to chemotherapeutic agents in non-small cell lung carcinoma, A549 lung cancer, hepatoma and pancreatic cancer cell lines [21,22]. Compounds including luteolin, trigonelline and triptolide showed improved activity against Nrf2 *in-vitro* and *in-vivo* [23,24]. Recent report from Peng et al., suggest that ethionamide suppression of ARE activity sensitised the monocytes to arsenic therapy [25]. Brusatol, a quassinoid extracted from *Brucea javanica* was recently identified to have potent inhibitory activity against Nrf2 protein levels ([26]) by enhancing its ubiquitination in a KEAP1 (Kelch-like ECH associated protein) independent mechanism. Even though the effect of Brusatol in inhibiting Nrf2 and improving chemosensitivity was well demonstrated in solid tumors, its role in hematological malignancies has not been evaluated.

The aim of the present study was to investigate the role of Nrf2 in resistance to Ara-C, Dnr, and ATO in AML and to demonstrate the effect of pharmacological inhibition of Nrf2 using Brusatol in modulating the chemoresistance.

## Materials and methods

### Drugs, chemicals, and antibodies

Cytosine β-D-arabinofuranoside hydrochloride (Ara-C HCl), Daunorubicin hydrochloride (Dnr HCl) and Methyl thiazolyldiphenyl-tetrazolium bromide (MTT) were obtained from Sigma-Aldrich (St Louis, MO, USA). Arsenic trioxide (ATO; Arsenox) was purchased from Intas Pharmaceuticals (Ahmedabad, India). Brusatol (NSC 172924-T/1) was provided by Developmental Therapeutics Program, National Cancer Institute (NCI-DTP, Bethesda, USA). All cell culture reagents were obtained from Thermo Scientific (Waltham, MA, USA). Rabbit polyclonal anti Nrf2 (H300, 1:200) was purchased from Santa Cruz Technologies (CA, USA). Rabbit monoclonal anti-Nrf2 (D1Z9C, 1:6400) for use in flowcytometry experiments, was purchased from Cell Signalling Technologies (Danvers, MA, USA), β Actin (AC-74, 1:5000) from Sigma-Aldrich (St. Louis, MO), rabbit polyclonal anti-KEAP1 from Cuz Bio (CSB-PA012147LA01HU) and horseradish peroxidase-conjugated goat anti-Rabbit (1:4000), goat anti-mouse IgG (1:20,000) secondary antibodies from Thermo Scientific (Waltham, MA, USA).

### Patient samples

Bone marrow samples were collected after obtaining written informed consent from patients with de-novo AML (excluding AML-M3). This proposal was approved by the Institutional Review Board (IRB Min No. 8312). Bone marrow mononuclear cells were isolated by Ficoll density gradient centrifugation.

### Cell lines and culture conditions

Human AML cell lines THP1 (National Centre for Cell Science, Pune, India), Molm13 (gift from Dr. Ellen Weisberg, Dana Farber Cancer Institute, Boston, MA, USA), U937 (gift from Dr. Stefan Glaser, Cancer and Hematology Division, Walter and Eliza Hall Institute of Medical Research, Australia) and HL60 (ATCC, CCL-240) were used in this study. Cells were cultured in RPMI media supplemented with Foetal bovine serum (10%) and antibiotics (100U/ml Penicillin and 100g/ml Streptomycin) in humidified atmosphere with 5% CO_2_ at 37°C. THP1 complete growth medium was prepared by supplementing 10% RPMI with β-mercaptoethanol (0.05 mM).

### RNA extraction and real-time quantitative PCR (RQ-PCR)

Total RNA was isolated using Tri-reagent (Sigma St. Louis, MO, USA) and the cDNA was prepared from 1μg of RNA using High Capacity cDNA Reverse Transcription Kit (Applied Biosystems, Foster City, CA, USA). Taqman^®^ Gene Expression Assays (Applied Biosystems, Foster City, CA) were used to analyze RNA expression of *NRF2* (Hs00975960_m1), Heme oxygenase (*HMOX-1*) (Hs01110250_m1), Glutathione cysteine ligase catalytic subunit (*GCLC*) (Hs00155249_m1), Glutathione cysteine ligase modifier subunit (*GCLM*) (Hs00157694_m1), NADPH Quinone oxidoreductase (*NQO1*) (Hs02512143_s1) and Glyceraldehyde 3-phosphate dehydrogenase (Hu *GAPDH*) (4352934_1101034) with TaqMan^®^ Universal PCR Master Mix (Applied Biosystems, Foster City, CA) in an 7500 Fast Real-Time PCR System (Life Technologies, Carlsbad, CA, USA). Relative gene expression (ΔCt) was calculated by normalizing individual gene expression to that of *GAPDH*. Fold-change expression values were provided as log-base 2 between control and the test groups. *KEAP1* RNA expression was analyzed in AML cell lines using SYBR Green chemistry (SYBR^®^ Premix Ex Taq^™^ II (Tli RNaseH Plus), TaKaRa Bio, Shiga, Japan) and (ΔCt) was calculated by normalizing individual gene expression to that of *β-Actin*. Primer sequences for *KEAP1* and *β*-*Actin* are listed in S1 Table.

### *In-vitro* cytotoxicity assay

Cells were seeded at a density of 10^5^ cells per well of a 96 well flat bottom plate and treated with increasing concentrations of Ara-C (0.1–80μM), Dnr (0.0025–1μM) or ATO (0.1–6μM). The plates were incubated for 48hrs in 5% CO_2_ at 37°C. Ten μl of MTT reagent was added to the wells, and the cell viability was measured at standard wavelength 570nm and reference wavelength 630nm using EL800 reader with KC junior software (Biotek Winooski, VT, USA). All the experiments were performed in triplicates. The combinatorial index (CI) was calculated using Compusyn software (ComboSyn, Inc., Paramus, NJ). CI = 1 indicates additive effect, CI<1 indicates synergism and CI > 1 indicates antagonism in drug combinations.

### Determination of Nrf2 expression and apoptosis by flow cytometry

Flow cytometric estimation of Nrf2 in cells was performed based on manufacturer's instruction (Cell Signaling). The cells were stained with rabbit primary monoclonal Nrf2 antibody and then incubated with anti- rabbit Alexa Fluor antibody (Molecular Probes, Eugene, OR). Apoptosis was determined by staining the cells with allophycocyanin (APC) conjugated Annexin V and 7-Amino-Actinomycin (7-AAD) (BD Pharmingen, San Diego, CA) based on manufacturer’s instruction. The redox status of cells was determined by incubating the cells with 2'-7'-Dichlorodihydrofluorescein diacetate DCFDA (10μM) (Sigma-Aldrich, St Louis, MO, USA) for 15min followed by measuring the mean fluorescent intensity (MFI). Flow cytometry analysis was performed using Accuri C6 cytometer (BD Biosciences, Franklin Lakes, NJ) with BD Accuri C6 Software (Version 1.0.264.21) and further analysed by flowJO.

### Nrf2 protein expression by western blot and subcellular localization by Immunofluorescence

Cells were washed with ice-cold PBS, and the whole cell lysates were prepared in Radioimmunoprecipitation assay (RIPA) buffer supplemented with protease inhibitor mixture (1X) (Roche Applied Science, IN, USA) and 2mM Phenyl methyl sulfonyl fluoride (Sigma-Aldrich) (Sigma–Aldrich, Bangalore, India). Protein concentration was estimated using Bradford method. Total proteins (50μg/lane) were resolved in 10% SDS-Polyacrylamide gel and transferred to polyvinylidene difluoride (PVDF) membranes. Membranes were blocked with 10% non-fat dry milk powder in TBS containing 0.1% Tween20 and then incubated with Anti-Nrf2 antibody. β –Actin was used as loading control. The bands were visualized using a chemiluminescence ECL system (Super Signal West Femto, Thermo Scientific). The intensity of protein bands was quantified by FluorChem E system using Digital Darkroom software. Subcellular localization of Nrf2 was determined by immunofluorescence as previously described [27].

### Preparation of nuclear lysate and determination of antioxidant response element (ARE) binding activity of Nrf2

Nuclear lysates were prepared from cell lines using nuclear extract kit (Active Motif Inc., Carlsbad, CA, USA). Protein concentration was estimated using Bradford method. Nrf2 binding to ARE was determined using TransAM Kit based on manufacturer’s instructions (Active Motif Inc., Carlsbad, CA, USA). Six micro gram of protein were added per well of 96 well plate to which the oligonucleotide has been immobilized. The spectrophotometric read out was taken at 450nm and represented as binding activity of Nrf2.

### Genomic DNA extraction and sequencing to identify mutations in KEAP1 gene

Genomic DNA was extracted from AML cell lines using phenol-chloroform method. Mutations in KEAP1 (exon 2 to exon 6) was screened by PCR followed by direct sequencing in an Applied Biosystems Genetic Analyzer 3130 (Foster City, CA, USA). The primer sequences used for PCR and sequencing are enlisted in S1 Table.

### Knockdown and overexpression of Nrf2

For generating doxycycline-inducible Nrf2 shRNA plasmid, validated shRNA for human Nrf2 (Sigma-Aldrich, St Louis, MO, USA; Cat. No: TRCN0000007555) was cloned into Plko.1 Tet-ON plasmid (Addgene clone ID 21915). This shRNA showed significantly better knock down of Nrf2 as reported previously in recent studies [28,29]. For generating Nrf2 overexpression plasmids, a lentiviral vector containing full-length Nrf2 cDNA, PLX304-Nrf2 (clone ID HsCD00445044), was purchased from DNASU plasmid repository (Biodesign Institute, Arizona State University, Arizona, USA).

Viral particles were generated by co-transfecting 293T cells with 2μg of PLKO.1-TET-ON-shNRF2 and 0.5μg each of helper plasmids -psPAX2 (Addgene plasmid no.12260), pMD2.G (Addgene plasmid no. 12259) using 3μl transfection reagent (Fugene 6, Promega, Madison, WI). The supernatant containing lentiviruses were collected at 24, 48, 72hrs, pooled, filtered and frozen at -80°C until use. Target cell lines (HL60, Molm13, U937, and THP1) were transduced with lentiviruses in the presence of 0.8μg/ml polybrene followed by spinfection to increase the transduction efficiency.

Nrf2 ShRNA transduced AML cells (THP1 and U937) were selected by puromycin (1.5μg/ml and 2μg/ml respectively), and the conditional knockdown of Nrf2 was achieved by the addition of doxycycline (1μg/ml). For cell lines transduced with full-length Nrf2 cDNA, cells were challenged with blasticidin S (2μg/ml for HL60 and 5μg/ml for Molm13) for seven days and blasticidin resistant cells were collected.

### Effect of brusatol treatment in combination with chemotherapeutic drugs on primary AML and normal BMMNCs

BMMNCs isolated from AML primary samples (n = 10) as well as normal healthy volunteers were incubated with 10nM of Brusatol followed by Ara-C, Dnr and ATO treatment for 48h. Cell viability was assessed by MTT assay and IC-50 was calculated.

### Clonogenicity by methylcellulose assay

THP1 cells (10X10^6^) incubated with and without Brusatol were seeded in methylcellulose (5X10^3^) (Stem cell Technologies, Vancouver, Canada), colonies were visualized and enumerated after ten days.

### Statistical analysis

Unpaired t-test, non-parametric t-test and ANOVA with Kruskal- Wallis correction were used where appropriate. All statistical analysis was done using GraphPad Prism software and p-value < 0.05 was considered as statistically significant.

## Results

### High Nrf2 expression is associated with chemoresistance to Ara-C, Dnr, and ATO in AML cell lines and primary AML cells

**Fig 1:**
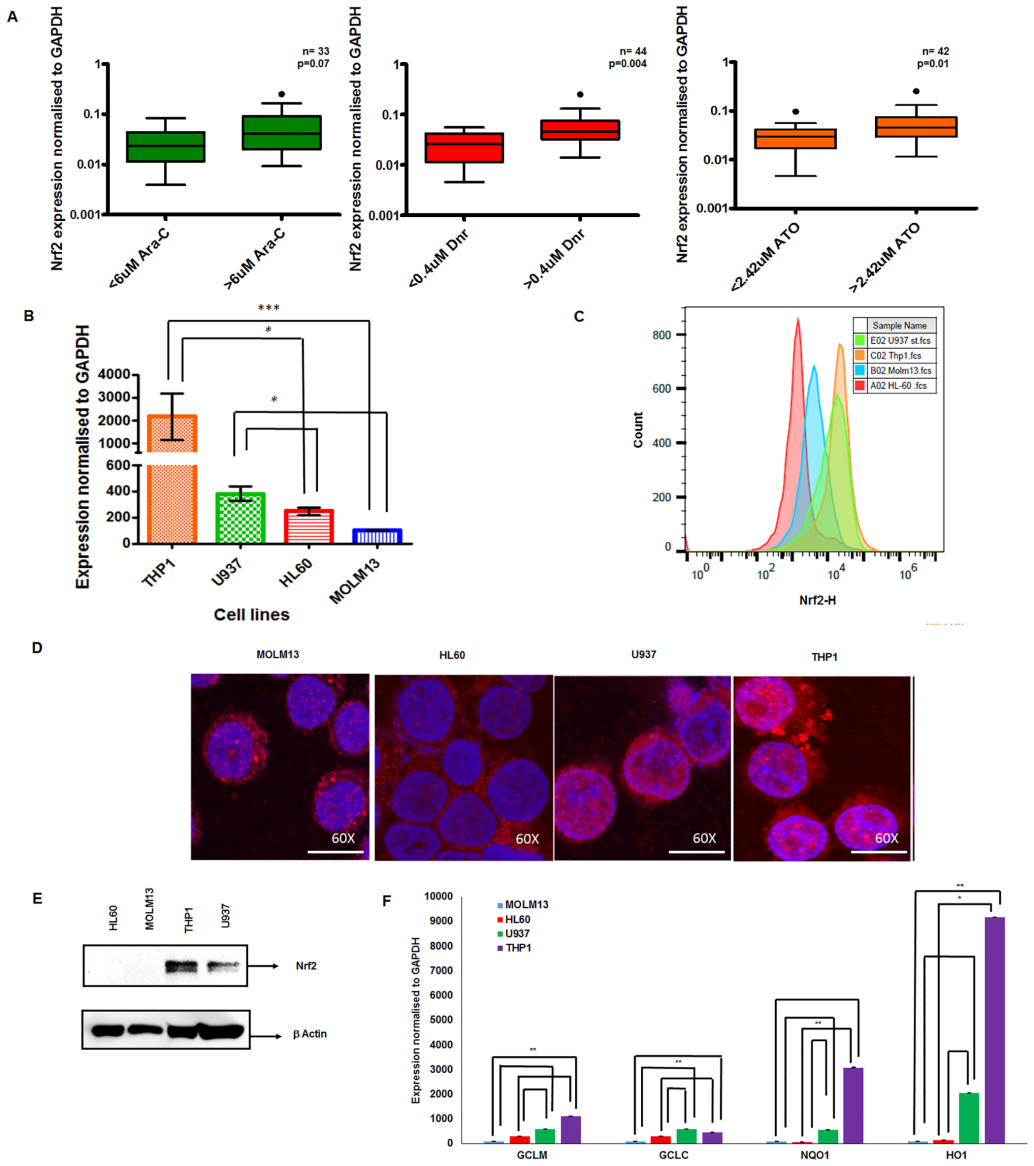
Primary AML cells and cell lines resistant to cytarabine, daunorubicin and arsenic trioxide show increased Nrf2 expression. **(A)** AML samples categorised based on median IC50 values to Ara-C (6μM), Dnr (0.4μM) and ATO (2.42μM) were analysed for the expression of *NRF2* by quantitative real time PCR. Y axis denotes relative expression (2^-dCT) of *NRF2* normalised to *GAPDH*. **(B)** *NRF2* levels in AML cell lines resistant (THP1, U937) or sensitive (HL-60, MOLM-13) to Ara-C, Dnr and ATO were analysed and represented as fold change (2^-ddCT) normalised to Molm13 (with low *NRF2* RNA)} (n = 5). *Statistical significance were calculated based on Kruskal–Wallis test and p<0.05 is indicated. **(C)** Flow cytometric analysis of total Nrf2 expression in AML cell lines (n = 4). **(D)** Immunofluorescence analysis of nuclear Nrf2 expression in AML cell lines {4′,6-diamidino-2-phenylindole (blue) and Nrf2 antibody (red) stained images overlay} (n = 2). **(E)** Immunoblotting of total Nrf2 in AML cell lines (β-actin was used as loading control) (n = 3). **(F)** Nrf2 downstream target expression {(*HO-1*, *NQO1*, *GCLC* and *GCLM*) normalized to *GAPDH* and expressed relative to that of MOLM-13 cell line (n = 4)}.*Statistical significance were calculated based on Kruskal–Wallis test and p<0.05 is indicated.

To understand the role of Nrf2 in chemoresistance, we compared the RNA expression of *NRF2* in primary AML cells to their *ex vivo* sensitivity to Ara-C, Dnr, and ATO. Primary AML samples were grouped according to their median IC50 values (Ara-C 6μM, Dnr 0.4μM and ATO 2.42μM). Primary AML samples with Ara-C, Dnr or ATO IC50 above the median had significantly higher *NRF2* RNA expression (p = 0.07, 0.004 and 0.01 respectively) compared to those below median (Fig 1A). Further, we compared the *in-vitro* sensitivity of AML cell lines to Ara-C, Dnr and ATO with *NRF2* RNA expression levels. Among the cell lines studied, THP1 and U937 with high IC50 values to all these three drugs also expressed high mRNA levels of *NRF2* compared to the cell lines Molm13 and HL60 with low IC50 values (Fig 1B). Flow cytometry analysis also revealed elevated intracellular protein levels of Nrf2 in THP1 and U937 compared to the sensitive cell lines HL60 and MOLM13 (Fig 1C & S1 Fig). Total and nuclear Nrf2 protein expression was higher in the resistant cell lines THP1 and U937 compared to Molm13 and HL60 as shown by immunoblot and immunofluorescence (Fig 1D and 1E). When the activation of Nrf2 mediated antioxidant response in these cells was investigated, the transcript levels of Nrf2 downstream targets *GCLC*, *GCLM*, *NQO1*, *HMOX-1* genes were high in resistant cell lines compared to the sensitive cell lines (Fig 1F).

### Nrf2 overexpression in AML cell lines is independent of *KEAP1* mutations and expression

As KEAP1 mutations are shown to be causing Nrf2 up-regulation, we screened for the presence of mutations in KEAP1 (exon 2-exon 6) in AML cell lines. We did not identify any mutation in the exonic regions of KEAP1 in all the cell lines screened **(data not shown)** suggesting that Nrf2 overexpression in AML is not due to KEAP1 mutations. We then compared the mRNA and protein expression of *KEAP1* in these cell lines. We observed that neither KEAP1 mRNA nor protein expression was significantly different between sensitive and resistant cell lines. (S2A and S2B Fig).

### Knockdown of Nrf2 sensitizes AML cell lines to Dnr and ATO by reducing the reactive oxygen scavenging capacity and down-regulation of target antioxidant genes

**Fig 2:**
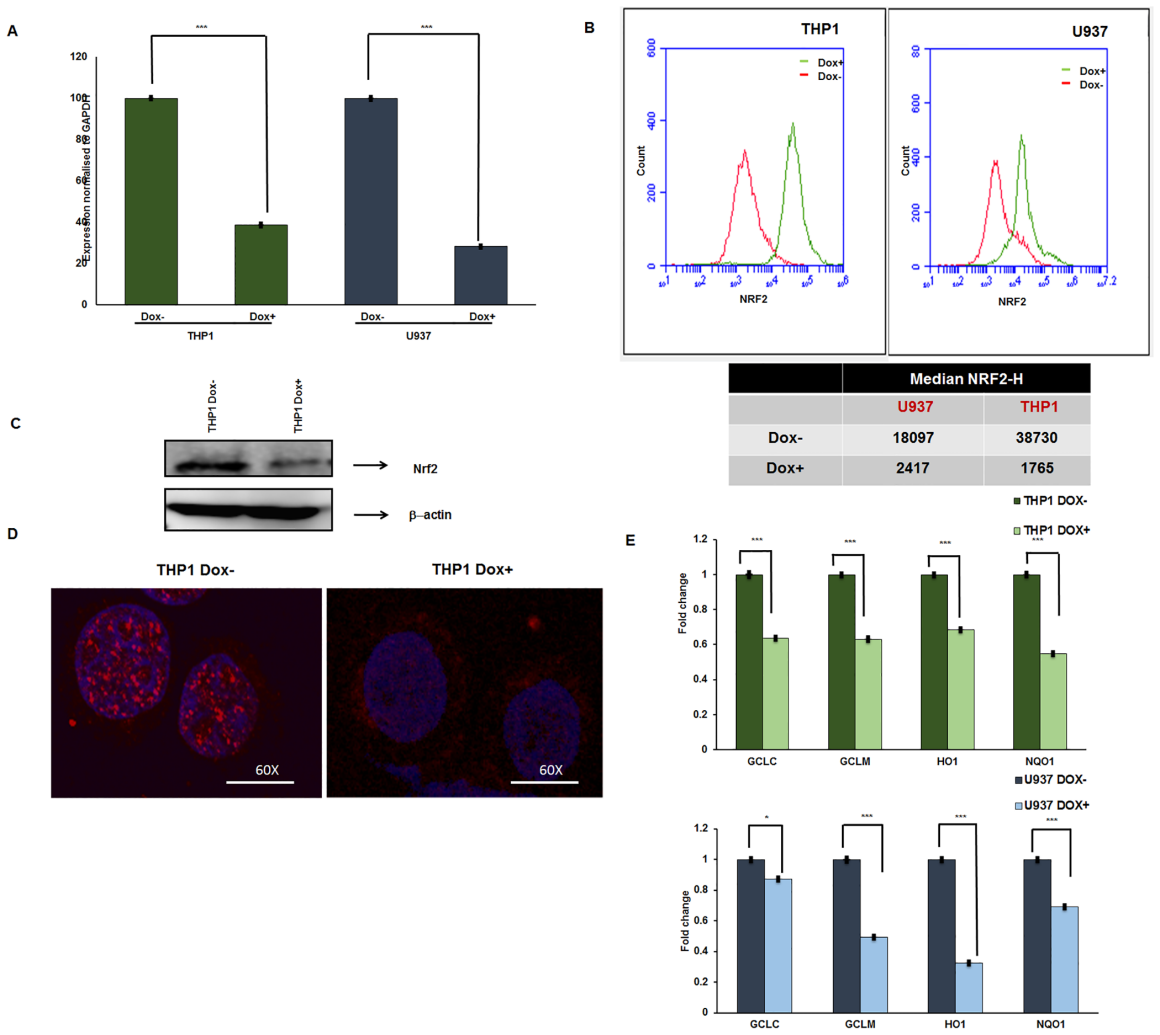
Conditional knockdown of *NRF2* reduces the total cellular and subcellular expression of Nrf2 as well as downstream target gene expression in AML cell lines. THP1 and U937 cells (with relatively high expression of Nrf2) were transduced with doxycycline-inducible Nrf2 shRNA system treated (+) or not treated (-) with Doxycycline. **(A)** RNA was extracted, and the expression of *NRF2* (normalized to *GAPDH* mRNA levels) was evaluated. Y axis represents *NRF2* expression (2^-ddCT) normalized to Dox- control cells (n = 4). *Statistical significance were calculated based on unpaired t test and p<0.05 is indicated. **(B)** Total protein levels of Nrf2 in THP1 and U973 (knockdown and control) cell lines was analysed by flow cytometry (n = 3). **(C)** THP1 cells were lysed, whole cell proteins were extracted, and 50μg of protein was resolved by SDS-PAGE; β—actin was used as loading control (n = 4) **(D)** subcellular localisation of Nrf2 was analysed by immunofluorescence {4′, 6-diamidino-2-phenylindole (blue) and Nrf2 antibody (red) stained images overlay} (n = 2) and **(E)** transcript levels of NRF2 downstream target genes (*HO-1*, *NQO1*, *GCLC and GCLM*) were analysed by real-time PCR. RNA expression of all target genes was normalized to *GAPDH* (n = 4). *Statistical significance were calculated based on unpaired t test and p<0.05 is indicated.

**Fig 3:**
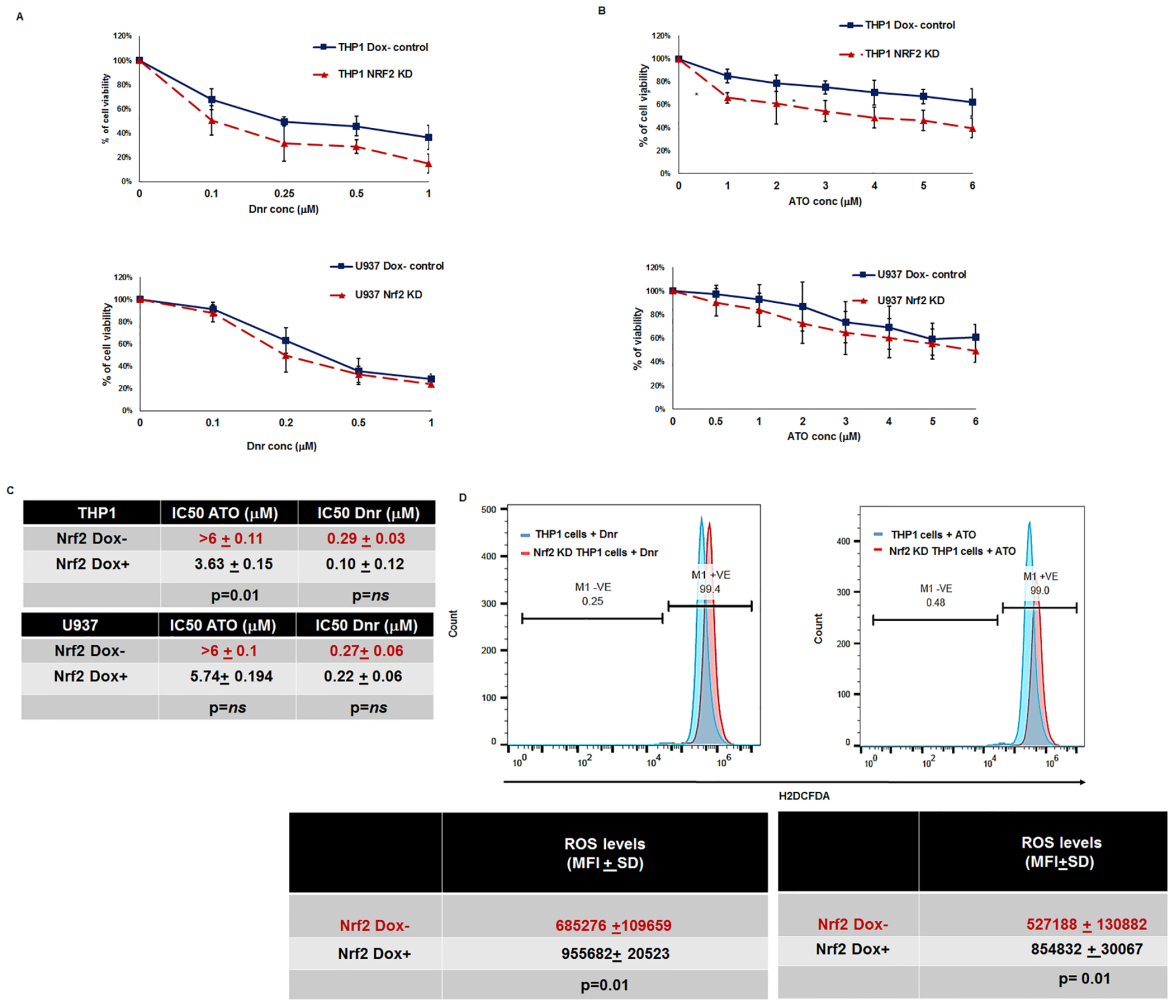
ShRNA knockdown of *NRF2* in AML cell line THP1 improved their sensitivity to Dnr, ATO and reduced IC50 by increasing ROS levels. THP1 and U937 cells (Dox+ and Dox-) were incubated with increasing concentration of Dnr **(A)** and ATO **(B)** for 48hrs and the *in-vitro* cytotoxicity was measured by MTT assay (n = 9). **(C)** IC-50 of Dox+ cells to Dnr and ATO was compared with Dox- cells. **(D)** THP1 cells were treated with Dnr (1μM) for 3hrs or ATO (6μM) for 6hrs, washed with PBS and incubated for 15 minutes with 10μM of H2DCFDA. ROS production was analyzed by flow cytometry (n = 3). Mean fluorescence (MFI) was calculated and compared with Dox- cells. *Statistical significance were calculated based on unpaired t test and p<0.05 is indicated.

To further investigate the role of Nrf2 in chemoresistance, we determined the effect of Nrf2 knockdown on sensitivity to Ara-C, Dnr, and ATO in the cell lines THP1, U937 with a high endogenous expression of Nrf2. As the constitutive knockdown of Nrf2 reduced the cell proliferation over time (as determined by trypan blue dye staining), we developed a conditional Nrf2 knockdown system. We did not observe any cell death (by trypan blue dye staining) or difference in the stages of cell cycle (S3A Fig upper panel) by the inducible knockdown system during the period of study. However, we observed nearly 30% apoptosis upon continuous long-term doxycycline exposure (S3A Fig lower panel). Using this system, we achieved 60–80% decrease in the Nrf2 RNA levels (Fig 2A) as well as total and subcellular protein expression (Fig 2B–2D) compared to the doxycycline negative control cells. As expected, we observed a concomitant decrease in the expression of Nrf2 downstream target genes *GCLC*, *GCLM*, *HMOX*-*1*, and *NQO1* (1.1, 1.6, 1.5 and 1.8 folds respectively) compared to Dox-control cells (Fig 2E). Doxycycline did not have any independent effect in reducing the Nrf2 levels as assessed by flow cytometry (S4 Fig). Knockdown of Nrf2 improved the *in-vitro* sensitivity of AML cell lines to Dnr (Fig 3A) and ATO (Fig 3B) as shown by their IC50 values (Fig 3C). However, we did not observe significant improvement in sensitivity to Ara-C in both THP1 and U937 cells compared to control cells (S5A Fig). Chemotherapeutic agents bring about cell kill by escalating the reactive oxygen species (ROS). When basal levels of Nrf2 is high, levels of ROS in cells does not cross the toxic threshold levels. We assessed whether Nrf2 knockdown compromises the cell’s ability to scavenge the ROS. Upon silencing of Nrf2 followed by treatment with chemotherapeutic agents (ATO and Dnr), we observed that accumulation of ROS was much higher in the knockdown cells compared to the control cells (Fig 3D) although only subtle increase in ROS levels was noted after treatment with Ara-C (S5B Fig).

### Pharmacological inhibition of Nrf2 brings down Nrf2 expression and induces early apoptosis in AML cells

**Fig 4:**
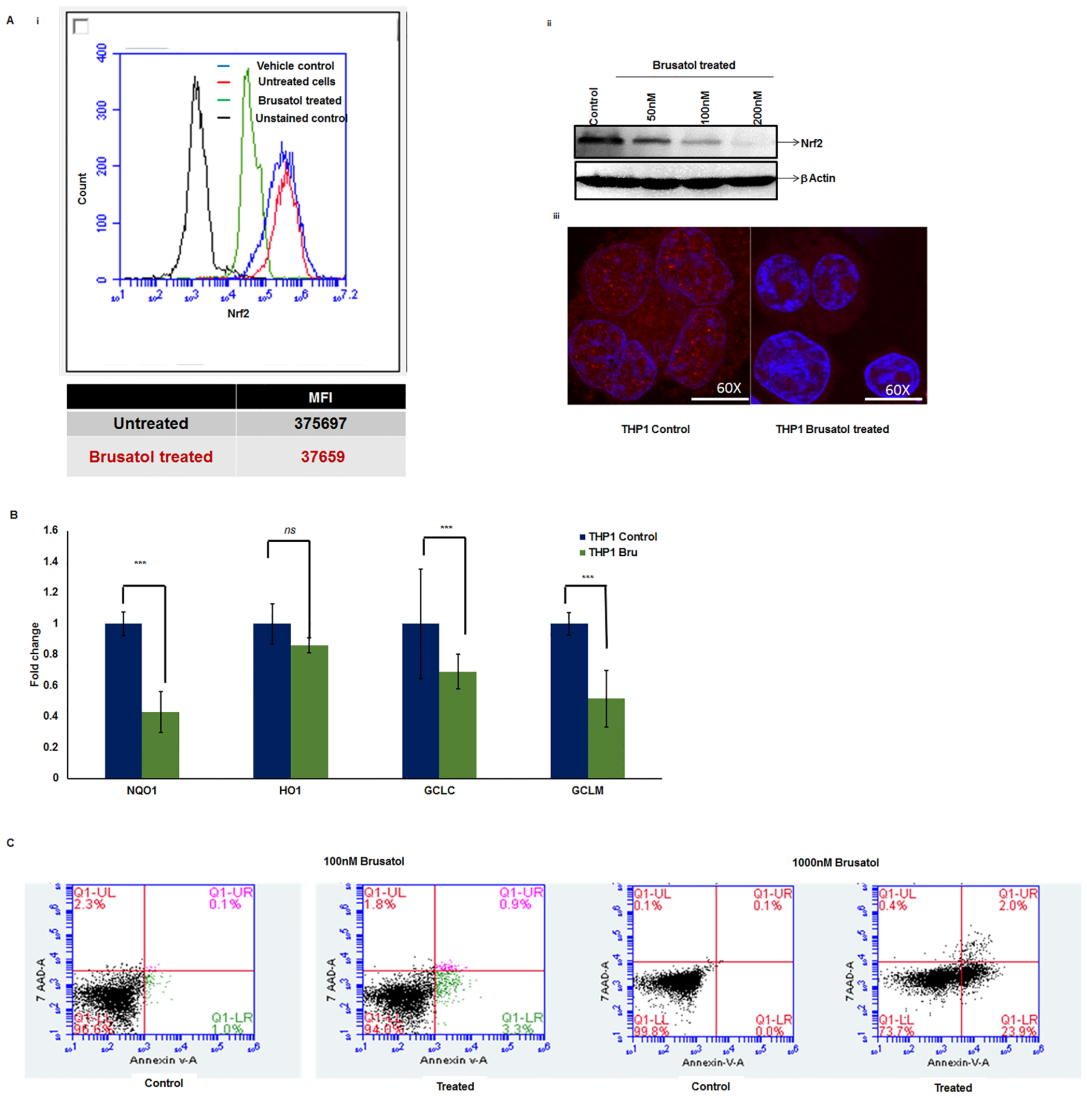
Brusatol treatment brings down Nrf2 expression and reduces colony forming capacity of THP1 cells. THP1 cells were treated with 1μM of brusatol reconstituted in DMSO (final concentration of 0.01%) for 6hrs. The expression of Nrf2 after treatment with brusatol was assessed by **(A)** flow cytometry (n = 2) (i), western blot with β—actin as the loading control (n = 3) (ii) and subcellular expression (iii). **(B)** THP1 cells were treated with 100nM of Brusatol for 6h and downstream targets of Nrf2 *GCLC*, *GCLM*, *HO-1*, *and NQO1* was evaluated by quantitative real time PCR (n = 4). RNA expression of all target genes was normalized to *GAPDH*. *Statistical significance were calculated based on unpaired t test and p<0.05 is indicated. **(C)** Apoptosis upon incubation with two different concentrations 100nM and 1000nM of Brusatol was assessed by Annexin V-7AAD.

**Fig 5:**
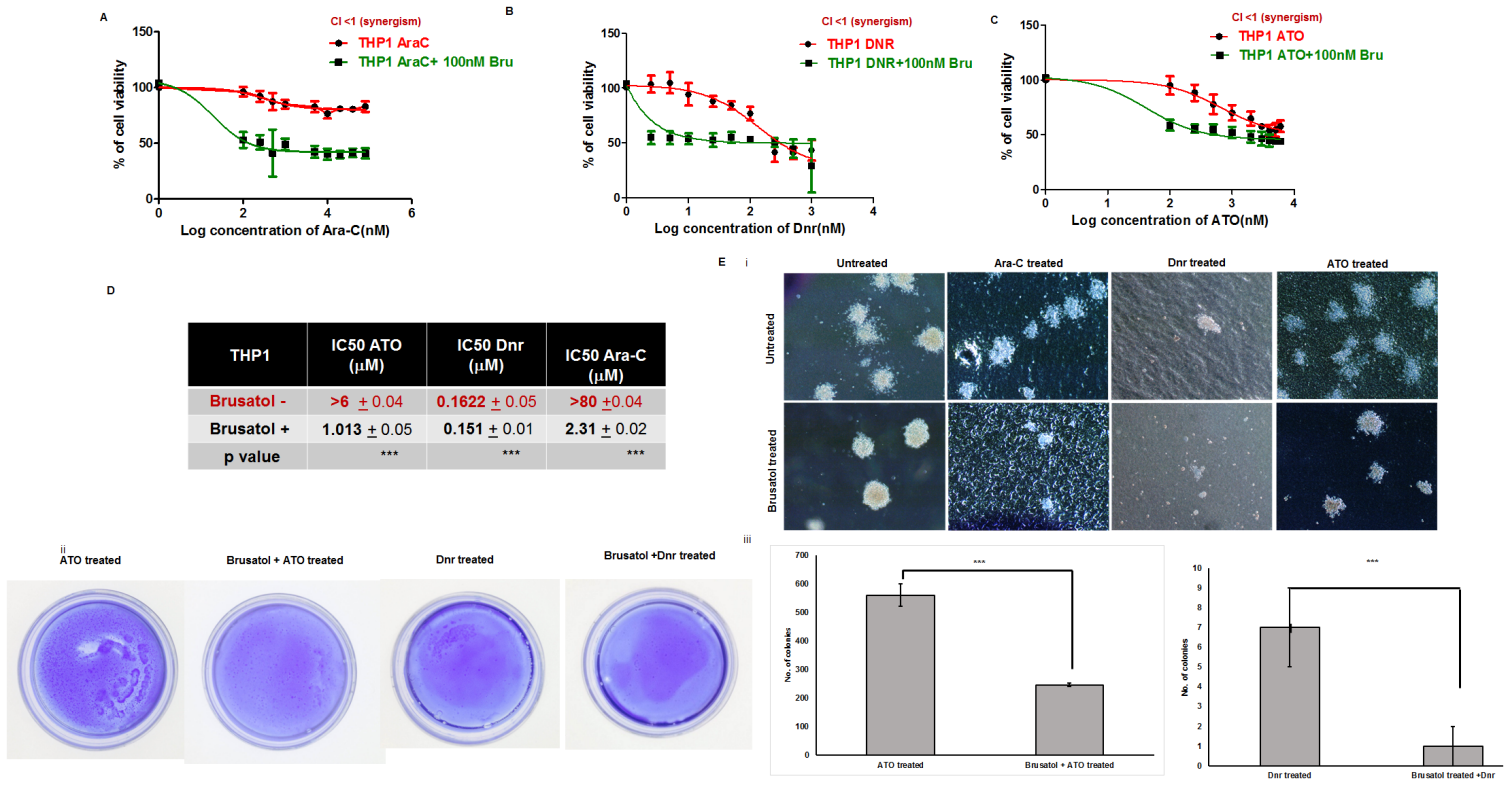
Pharmacological inhibition of Nrf2 with brusatol brings down the IC50 of Ara-C, Dnr & ATO in resistant AML cell lines. THP1 cells were incubated with Nrf2 inhibitor Brusatol 100nM for 6hrs, followed by increasing concentration of **(A)** Ara-C (0.1–80μM), **(B)** Dnr (0.0025–1μM) and **(C)** ATO (0.1–6μM) for 48hrs. *In-vitro* cytotoxicity was measured by MTT assay (n = 9). **(D)** IC50 of THP1 to Dnr and ATO was compared with untreated control cells. **(E)** THP1 cells were incubate with 100nM of Brusatol followed by treatment with 5μM of Ara-C, 0.25μM of Dnr or 6μM of ATO for 24hrs. Control and treated (5*10^3^) cells were seeded in methyl cellulose medium (n = 2) and enumerated on day 14. Light microscopic images of colonies were taken on day 14 (10X magnification) (i). Control or treated (5*10^3^) cells seeded in methyl cellulose medium were stained with methylene blue (ii) and colonies were enumerated (n = 2)(iii). Brusatol alone minimally reduces the colony forming capacity of THP1 cells and considerably reduces in combination with Ara-C, Dnr and ATO.

To determine the effect of pharmacological inhibition of Nrf2 in modulating chemoresistance, we tested the combinatorial effect of Nrf2 inhibitors with Ara-C, Dnr, and ATO. Among the compounds screened for inhibitory activity against Nrf2 (S6 Fig), brusatol was found to be the most efficient inhibitor of Nrf2 (Fig 4A). The effect of this compound in reducing the elevated Nrf2 levels in a dose and time-dependent manner was assessed in THP1 cell line. We observed a considerable reduction in total and subcellular Nrf2 levels with brusatol treatment (Fig 4A). As Nrf2 activation of antioxidant enzymes is achieved through their binding with the antioxidant response elements (ARE), we evaluated the ARE binding activity of Nrf2 after treatment with Brusatol. We observed that in THP1 cell line, ARE binding activity decreased moderately upon treatment with Brusatol similar to that of Nrf2 knock down THP1 cells (S7A Fig). While treatment with chemotherapeutic agents (Ara-C, Dnr and ATO) increased the ARE binding activity of Nrf2 (S7B–S7D Fig) and thereby the expression of Nrf2 downstream target (S7E Fig), treatment with brusatol followed by administration of chemotherapeutic agents reduced the ARE binding of Nrf2. Among them, the binding activity was considerably reduced with Dnr but to a lesser extent with Ara-C and ATO treatment. As expected, Nrf2 inhibition by brusatol also reduced the transcript levels of Nrf2 downstream targets *GCLC*, *GCLM*, *HMOX-1*, and *NQO1* (by 1.44, 1.93, 1.16 and 2.33 folds respectively) compared to the control (Fig 4B). To determine the effect of this compound on apoptosis, the cells were incubated with 1000nM and 100nM brusatol for 16hrs followed by Annexin V 7AAD measurement. We observed that brusatol at low concentration (100nM) did not bring about apoptosis. Moreover, at higher concentration Brusatol (1000nM) had minimal effects in bringing about early apoptosis (23.7%) and no effects on late apoptosis (Fig 4C) (S8 Fig). When tested for the effect of pharmacological inhibition of Nrf2 on chemosensitivity of AML cell lines, primary AML cells as well as normal BMMNCs, we observed that co-administration of brusatol in resistant AML cell lines expressing high levels of *NRF2* (THP1 and U937) followed by ATO, Ara-C and Dnr improved their sensitivity to these drugs (Fig 5A–5D and S9A–S9C Fig). Majority of the primary AML samples, showed improved sensitivity to treatment with chemotherapeutic agent when brusatol was co-administered (S2 Table). However, brusatol had no detrimental effect in cell viability in normal donor cells as assessed by *in-vitro* cytotoxicity. To further test the effect of brusatol in bringing down the colony forming capacity of AML cells, brusatol treated and untreated cells were plated on methylcellulose medium. We observed that brusatol as a single agent reduced the colony numbers compared to control (480±10 vs. 600±11). Even though ATO, Ara-C or Dnr treatment alone reduced the colony number (to 560+40, 481+20 and 7±2 respectively), in combination with brusatol colonies were further reduced (246+6, 311+5 and 1+1 respectively) (Fig 5E).

### Overexpression of NRF2 induces resistance of AML cells to Ara-C, Dnr, and ATO and attenuates their sensitivity to Nrf2 inhibitors

**Fig 6:**
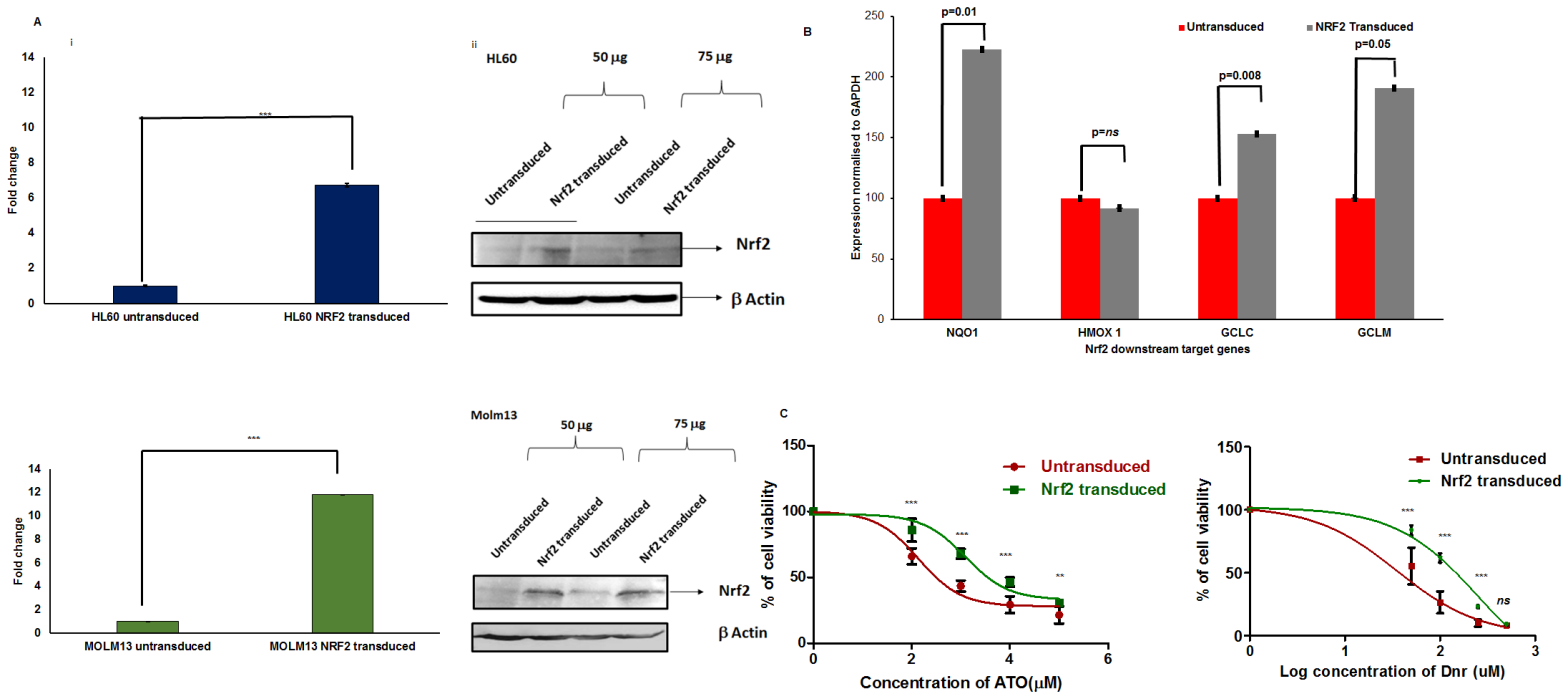
*NRF2* overexpression in AML cell lines increases IC50 of ATO & Dnr by up-regulating downstream target expression. Two AML cell lines (HL-60 and MOLM-13) with relatively low expression of *NRF2* were made to stably over express *NRF2*. **(A)** Overexpression was further confirmed by quantitative real-time PCR (i) and western blot (ii) for Nrf2 **(B)** Nrf2 downstream target genes (*GCLC*, *GCLM*, *NQO1*, *and HMOX-1*) were analyzed by real time quantitative PCR. **(C)** *In-vitro* cytotoxicity of overexpressed cells to Dnr and ATO was compared with control cells. Overexpression increases resistance to Dnr and ATO.

To further establish the specific role of Nrf2 in chemoresistance to Ara-C, Dnr, and ATO, *NRF2* was overexpressed in AML cell lines using lentiviral expression system. Overexpression was confirmed by immunoblot and RQ-PCR. We observed an increase in the levels of Nrf2 both at the transcript and protein levels compared to the control (Fig 6A). Expression of Nrf2 downstream targets, *GCLC*, *GCLM*, *NQO1* except *HMOX*-*1* was found to be elevated in over-expressed cells (Fig 6B). Constitutive Nrf2 overexpression also increased resistance to ATO and Dnr as indicated by increased cell viability (Fig 6C).

## Discussion

Nrf2 is a redox-dependent transcription factor regulating several antioxidants and phase II detoxifying genes thereby protecting the cells from oxidative or electrophilic insults. Previous reports in AML, and other solid tumors have shown that Nrf2 is associated with resistance to chemotherapeutic agents [30]. Although the previous studies have compared the Nrf2 expression in AML cells with normal CD34+ cells, they have not compared Nrf2 levels within the AML population. In primary AML samples at diagnosis, we observed that samples with IC50 values to Ara-C, Dnr, and ATO above the median had high *NRF2* mRNA expression compared to those below the median suggesting that Nrf2 is aberrantly expressed in resistant AML samples.

In this study, we explored the mechanisms behind Nrf2 upregulation in AML samples. As mutations in KEAP1 gene have been shown to result in upregulation of Nrf2 as shown in several solid tumors [31], we screened for KEAP1 mutations in AML cell lines. We did not detect any mutations in the KEAP1 gene which was in concordance with the findings from Rushworth et al., [32]. The mRNA and protein expression of KEAP1 were also not significantly different between sensitive and resistant cell lines. Hence, we looked at KEAP1 independent mechanisms of Nrf2 activation. As PI3/AKT/GSK axis being a prominent pathway inducing Nrf2 activation [33] and as Akt inhibitor, MK2206 has been shown effective in reducing Nrf2 accumulation in A549 lung carcinoma cells [34], we tested the effect of Nrf2 inhibition with MK2206 in AML cells. However, in AML cells even at pharmacologically relevant concentration of MK2206, we did not find any considerable reduction in Nrf2 levels, suggesting that unlike in solid tumors, Nrf2 regulation in AML is not mediated via PI3/Akt pathway.

To further understand the mechanistic role of Nrf2 in chemoresistance, gene-specific knockdown of Nrf2 was carried out. Unlike previous studies which have attempted transient knockdown, we used much efficient constitutive system of knockdown of Nrf2 [35,36]. The efficiency of knockdown in HeLa and MCF7 cells using this method resulted in complete absence of Nrf2 protein expression. In contrast to solid tumors, Nrf2 shRNA transduction in AML cells brought about increased cell death which was efficiently rescued by inducible knockdown suggesting that Nrf2 provides survival advantage to AML cells. These results are similar to that of Peng et al [25], although these authors used a stable constitutive knockdown of *NRF2* unlike that of ours which is an inducible system.

Previous studies have reported that knockdown of Nrf2 sensitized AML cells to Ara-C and Dnr in combination. However, we evaluated the role of Nrf2 in sensitizing cells individually to the drugs Ara-C, Dnr, and ATO. We observed that silencing Nrf2 sensitized AML cells to Dnr and ATO but not so much to Ara-C. Also, the escalation of ROS levels upon Nrf2 knockdown was less in Ara-C treated cells. These findings suggest that Nrf2 plays relatively minimal role in Ara-C resistance than to Dnr and ATO.

Pharmacological inhibition of Nrf2 using flavonoid compounds have been tried in several tumors [37], but limited reports are available with respect to AML [23,25]. Here we have used brusatol which has been shown in solid tumors to significantly improve sensitivity to doxorubicin, oxaliplatin, and bleomycin by reducing the ARE activity [22]. Among the various pharmacological Nrf2 inhibitors screened, including MK2206, Luteolin, and U0126, brusatol showed maximum inhibition of Nrf2 in AML cells. Luteolin having shown to reduce ARE activity in NSCLC was tested for its effect in AML cell lines. Luteolin did not reduce Nrf2 expression considerably but independently reduced cell viability of FLT3-ITD mutated (Molm13, Molm-14, and MV4-11), and c-kit mutated (Kasumi-1) AML cell lines by ~50%. Even though MK2206 treatment did not significantly reduce Nrf2 expression, this resulted in increased cytotoxicity in FLT3-ITD mutated cell lines as expected.

Apart from their role in improving chemosensitivity, brusatol also enhanced the potential of cisplatin to reduce the colony formation in A549 lung cancer cells [26]. Similar to this observation, brusatol treatment in AML cells either alone or in combination with chemotherapeutic agents considerably reduced colony forming capacity of these cells. However, the mechanism of action of brusatol in improving chemoresistance in AML needs to be further explored.

As Nrf2 is proven to play a pivotal in chemoresistance, we have assessed the effect of overexpression of Nrf2 on the chemoresistance to Ara-C, ATO, and Dnr in AML cell lines. We observed in Nrf2 overexpressed cell lines an increased functional effect is achieved only after stabilization of Nrf2 using the compound, tert butinyl hydroxyl quinone (tBHQ). This suggests that apart from activation, stabilization of Nrf2 determines its function. Although brusatol was shown to reduce Nrf2 protein levels, its mechanistic role and toxicity profile remains unclear. Also, the efficacy of the drug needs to be further demonstrated with *in-vivo* models. The efficacy of Nrf2 inhibitors like triptolide in reducing LSC like cells was shown to have better therapeutic potential in treating relapsed and refractory AML [23]. Even though this study shows a possible role of the Brusatol in reducing colony formation capacity of AML cells, we did not address their role in specific to leukemic stem cells (CD34+38-) in AML. Another major limitation of this study is that all the experiments were performed in AML cell lines and primary AML cells while this study would greatly benefit from evaluation of single and combination therapy in mouse models of AML.

In conclusion, our study demonstrates that Nrf2 could be an ideal druggable target in AML, more so to the drugs that function through ROS. This study suggests the possibility of using Nrf2 inhibitors like brusatol in combination with chemotherapeutic agents to modulate drug resistance in AML.

## Supporting information

S1 Fig Total protein level Nrf2 in AML cell lines were measured by flow cytometry. Cells were fixed and permeabilized and incubated with Nrf2 antibody. Cells were then stained with a fluorochrome-tagged secondary antibody, and fluorescence intensity was measured. Values represent mean ± SD of four independent experiments. Fluorescence intensity of resistant cell lines THP1 and U937 was compared with sensitive cell lines HL60, MOLM13, and statistical significance was determined by Kruskal Wallis test. *Statistical significance (p<0.05) is indicated.
(TIF)

S2 Fig KEAP1 expression in AML cell lines. Keap1 expression at protein level (A) and mRNA level (B) were not significantly different between resistant and sensitive AML cell lines. Keap1 RNA expression was normalised to β-Actin and expressed in dCT, where higher the dCt lower the expression and vice versa.
(TIF)

S3 Fig shRNA knockdown of *NRF2* in AML cell line THP1 did not affect cell cycle initially but increased the apoptosis at later time periods. (A)THP1 cells after *NRF2* knockdown was incubated with PI for 15min. G1/S/G2M was analyzed in control and knockdown cells. Sub G0 phase which relates to apoptosis was measured and compared with Dox-control cells.
(TIF)

S4 Fig Doxycycline did not have any independent effect in reducing the Nrf2 levels. THP1 and U937 cells (1*10^6^) were treated with Doxycycline (1μg/ml) for 24h and Nrf2 expression was determined by flow cytometry. The Nrf2 expression levels were compared with untreated cells.
(TIF)

S5 Fig shRNA knockdown of NRF2 in AML cell line THP1 and U937 did not significantly improve their sensitivity to Ara-C. shRNA knock down of NRF2 in THP1 cells showed reduced ROS levels compared to control cells. **(**A) *The in-vitro* sensitivity of knockdown cells to Ara-C was measured by MTT assay in THP1 (upper panel) and U937 (lower panel). **(B)** THP1 cells were incubated with 5μM of Ara-C for 6hrs and washed with PBS, incubated for 15 minutes with 10μM of H2DCFDA. ROS production was analyzed by flow cytometry.
(TIF)

S6 Fig U0126 (MEK inhibitor), MK2206 (Akt inhibitor) and luteolin does not considerably bring down Nrf2 expression. AML cell line THP1 was treated with (A) 10μM of MK2206 (B) 10μM of U0126 or (C) 40μM of Luteolin for 24hrs and expression of Nrf2 was measured by flow cytometry.
(TIF)

S7 Fig Brusatol reduced the ARE binding activity of Nrf2 which was increased upon treatment with chemotherapeutic agents. AML cell line THP1 was treated with and without 100nM of Brusatol for 6h. This was followed by treatment with Ara-C (5μM), Dnr (1μM) and ATO (6μM) for another 24h. Nuclear lysates were quantified and 6μg of protein was added per well. Nuclear lysates were also prepared from Nrf2 knock down THP1 cells. ARE binding activity was determined spectrophotometrically at 450nm. **(A)** Brusatol effectively reduced the ARE binding activity of Nrf2; similar effect was observed in Nrf2 knock down THP1 cells. Treatment of THP1 cells with chemotherapeutic agents Ara-C **(B)**, Dnr **(C)** and ATO **(D)** increased the ARE activity as well as expression of downstream targets **(E),** while Brusatol co treatment reduced this activity. Brusatol reduced ARE activity moderately in Dnr and minimally in ATO and Ara-C treated cells.
(TIF)

S8 Fig Brusatol at high concentration induced early apoptosis in THP1 cells. THP1 cells were treated with two different concentrations of Brusatol (100nM & 1000nM) and incubated for 6hrs. After incubation, cells were washed and stained with Annexin V 7AAD and the apoptosis was measured. Values represent mean ± SD of two independent experiments.
(TIF)

S9 Fig Pharmacological inhibition of Nrf2 using brusatol brings down the IC50 of Ara-C, Dnr & ATO in U937 cell line. U937 cells were incubated with Nrf2 inhibitor Brusatol 100nM for 6hrs, followed by increasing concentration of **(A)** Ara-C, **(B)** Dnr and **(C)** ATO for 48hrs. *In-vitro* cytotoxicity was measured by MTT assay.
(TIF)

S1 Table List of primers used for Nrf2 and Keap1 sequencing. (TIF)

S2 Table Brusatol sensitized AML primary cells to Ara-C, Dnr and ATO. Primary samples at diagnosis was subjected to pre-treatment with brusatol followed by increasing concentrations of **(A)** Ara-C (0.1–80μM), **(B)** Dnr (0.0025–1μM) and **(C)** ATO (0.1–6μM) for 48h. *Ex -vivo* cytotoxicity was measured by MTT assay.
(TIF)

## Abbreviations

AML: Acute Myeloid leukemia
Ara-C: Cytarabine
ATO: Arsenic trioxide
Dnr: Daunorubicin
FLT3: Fms like tyrosine kinase
GCLC: Glutamate-cysteine ligase catalytic subunit
GCLM: glutamate-Cysteine Ligase Modifier Subunit
HMOX1 or HO1: Heme oxygenase 1
KEAP1: Kelch like ECH associated protein
NQO1: NADPH Quinone oxidoreductase
NRF2: NFE2-related factor 2
ROS: reactive oxygen species
