# Supplementary figures and images for: Role of NF-E2 related factor 2 (Nrf2) on chemotherapy resistance in acute myeloid leukemia (AML) and the effect of pharmacological inhibition of Nrf2
Source: PLoS One. 2017 May 15;12(5):e0177227. doi: 10.1371/journal.pone.0177227 (PMC5432104; doi:10.1371/journal.pone.0177227)

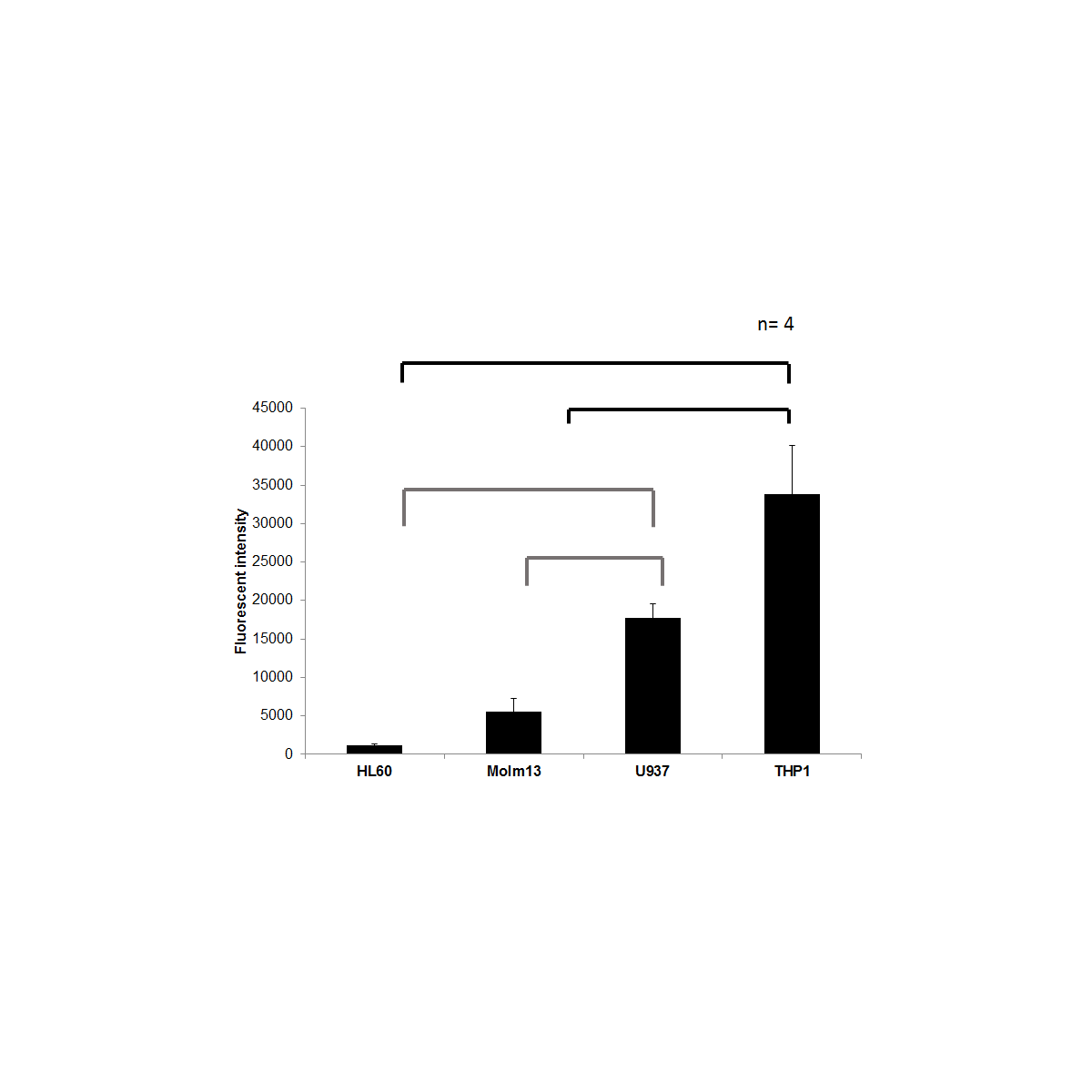

Supplement: S1 Fig — Cells were fixed and permeabilized and incubated with Nrf2 antibody. Cells were then stained with a fluorochrome-tagged secondary antibody, and fluorescence intensity was measured. Values represent mean ± SD of four independent experiments. Fluorescence intensity of resistant cell lines THP1 and U937 was compared with sensitive cell lines HL60, MOLM13, and statistical significance was determined by Kruskal Wallis test. *Statistical significance (p<0.05) is indicated. (TIF) [file pone.0177227.s001.tif]

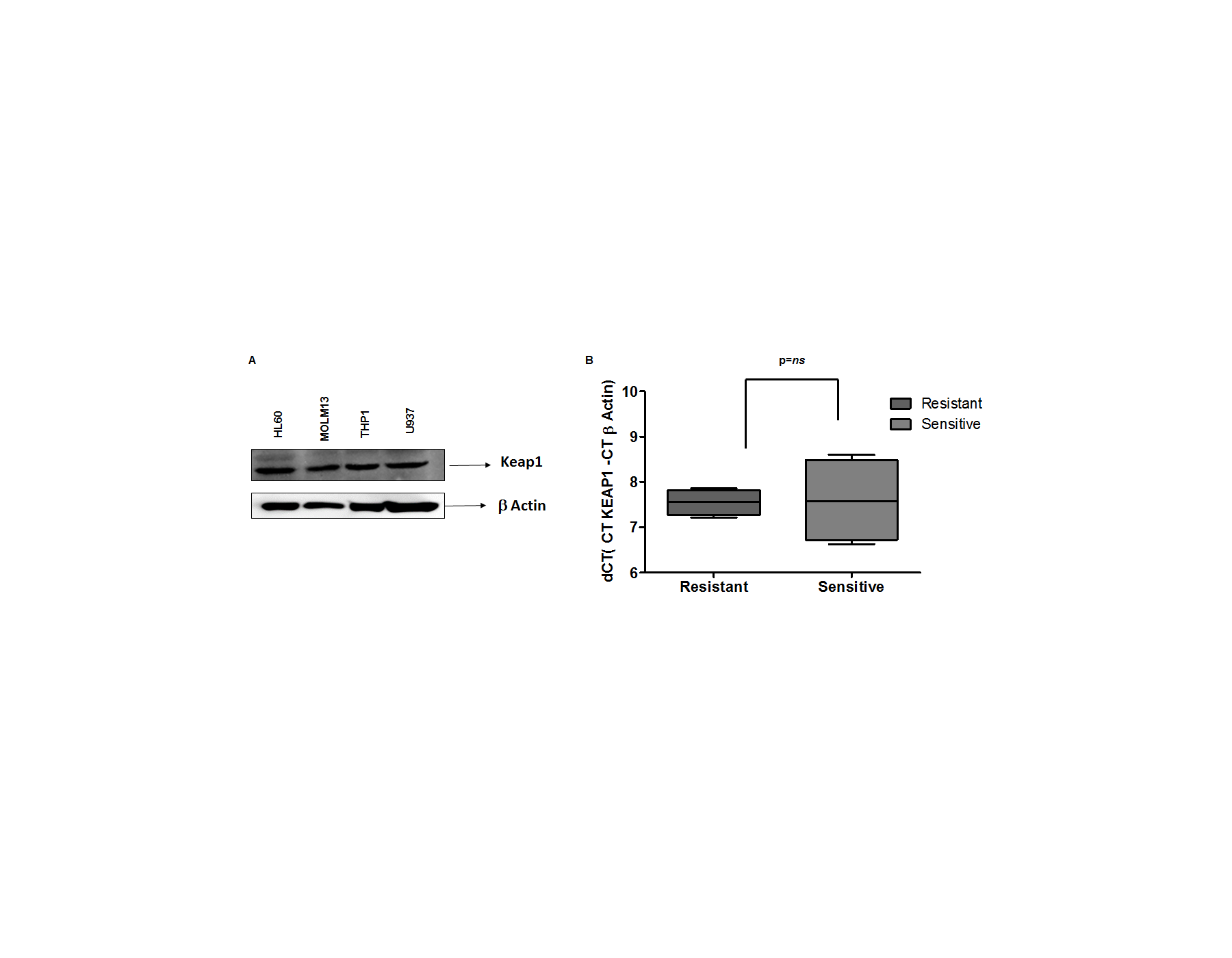

Supplement: S2 Fig — Keap1 expression at protein level (A) and mRNA level (B) were not significantly different between resistant and sensitive AML cell lines. Keap1 RNA expression was normalised to β-Actin and expressed in dCT, where higher the dCt lower the expression and vice versa. (TIF) [file pone.0177227.s002.tif]

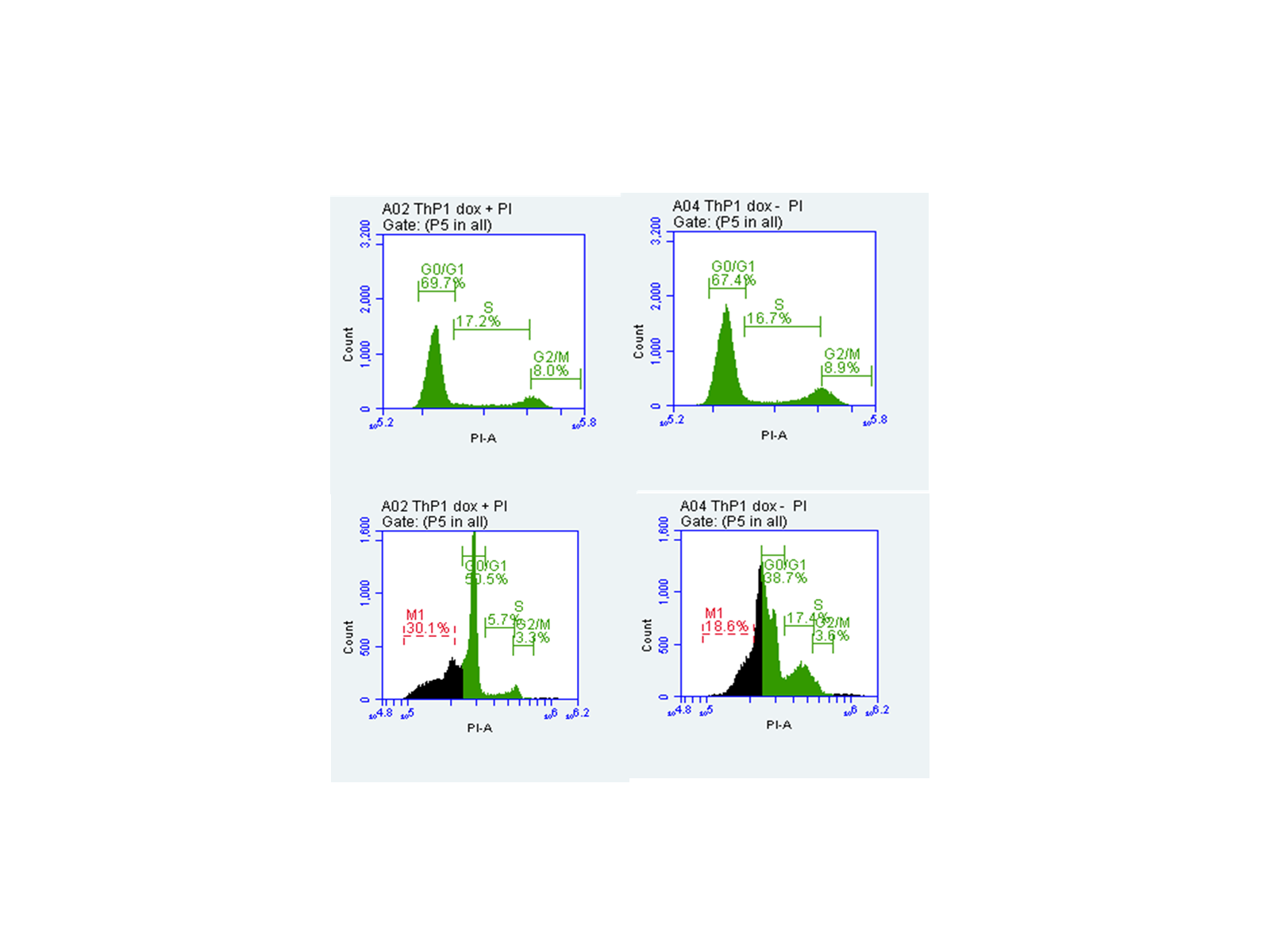

Supplement: S3 Fig — (A)THP1 cells after NRF2 knockdown was incubated with PI for 15min. G1/S/G2M was analyzed in control and knockdown cells. Sub G0 phase which relates to apoptosis was measured and compared with Dox-control cells. (TIF) [file pone.0177227.s003.tif]

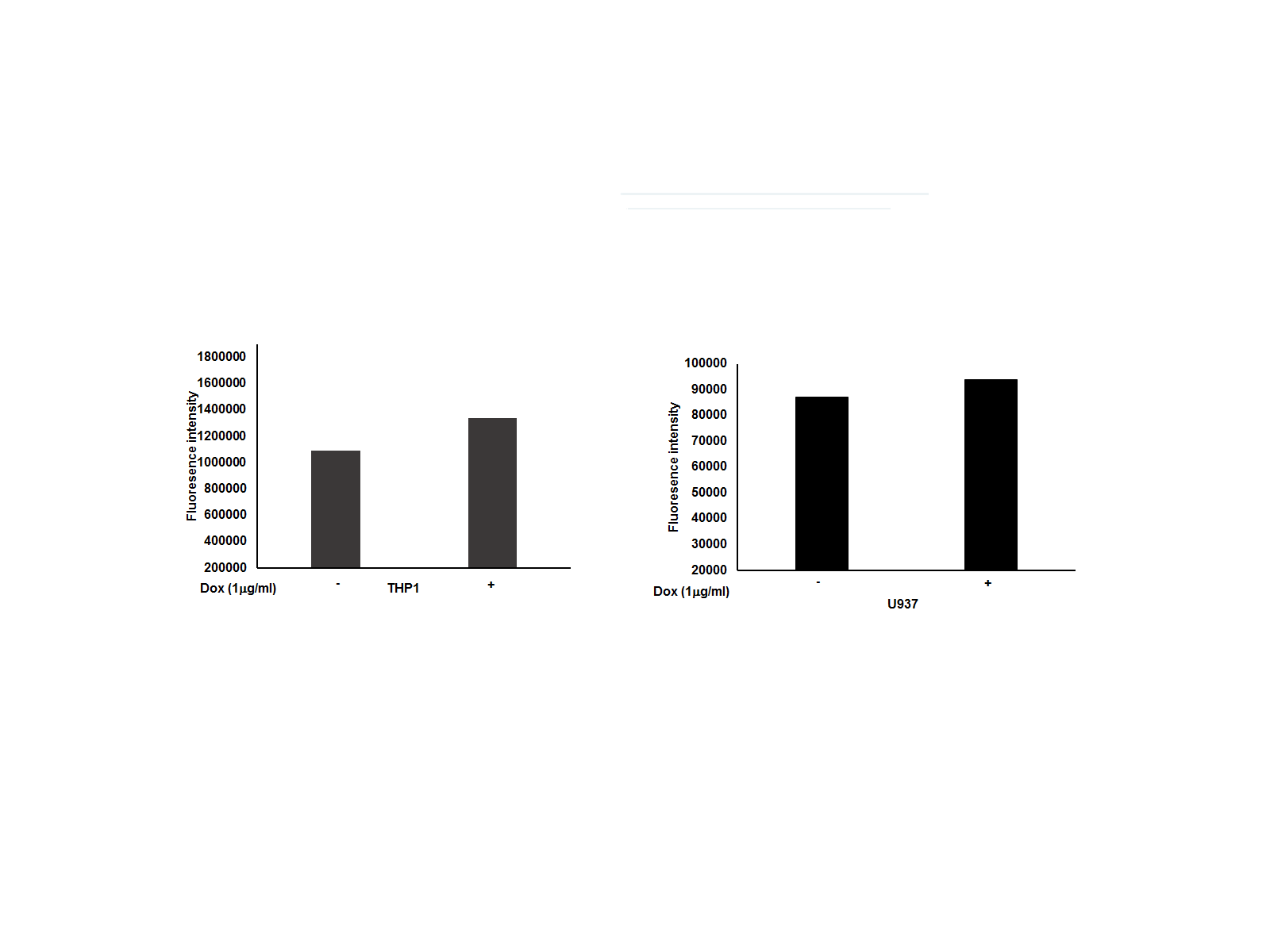

Supplement: S4 Fig — THP1 and U937 cells (1*106) were treated with Doxycycline (1μg/ml) for 24h and Nrf2 expression was determined by flow cytometry. The Nrf2 expression levels were compared with untreated cells. (TIF) [file pone.0177227.s004.tif]

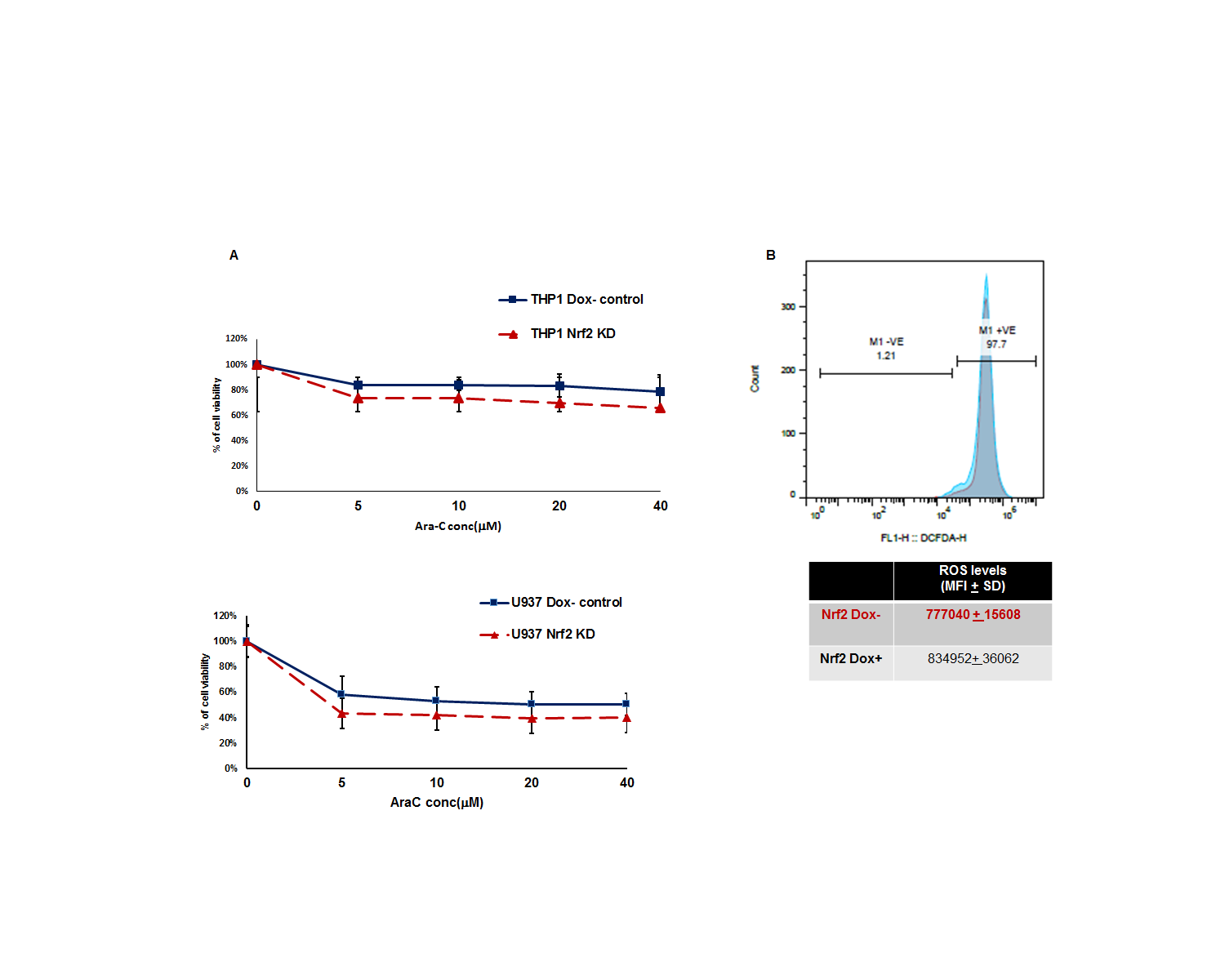

Supplement: S5 Fig — (A) The in-vitro sensitivity of knockdown cells to Ara-C was measured by MTT assay in THP1 (upper panel) and U937 (lower panel). (B) THP1 cells were incubated with 5μM of Ara-C for 6hrs and washed with PBS, incubated for 15 minutes with 10μM of H2DCFDA. ROS production was analyzed by flow cytometry. (TIF) [file pone.0177227.s005.tif]

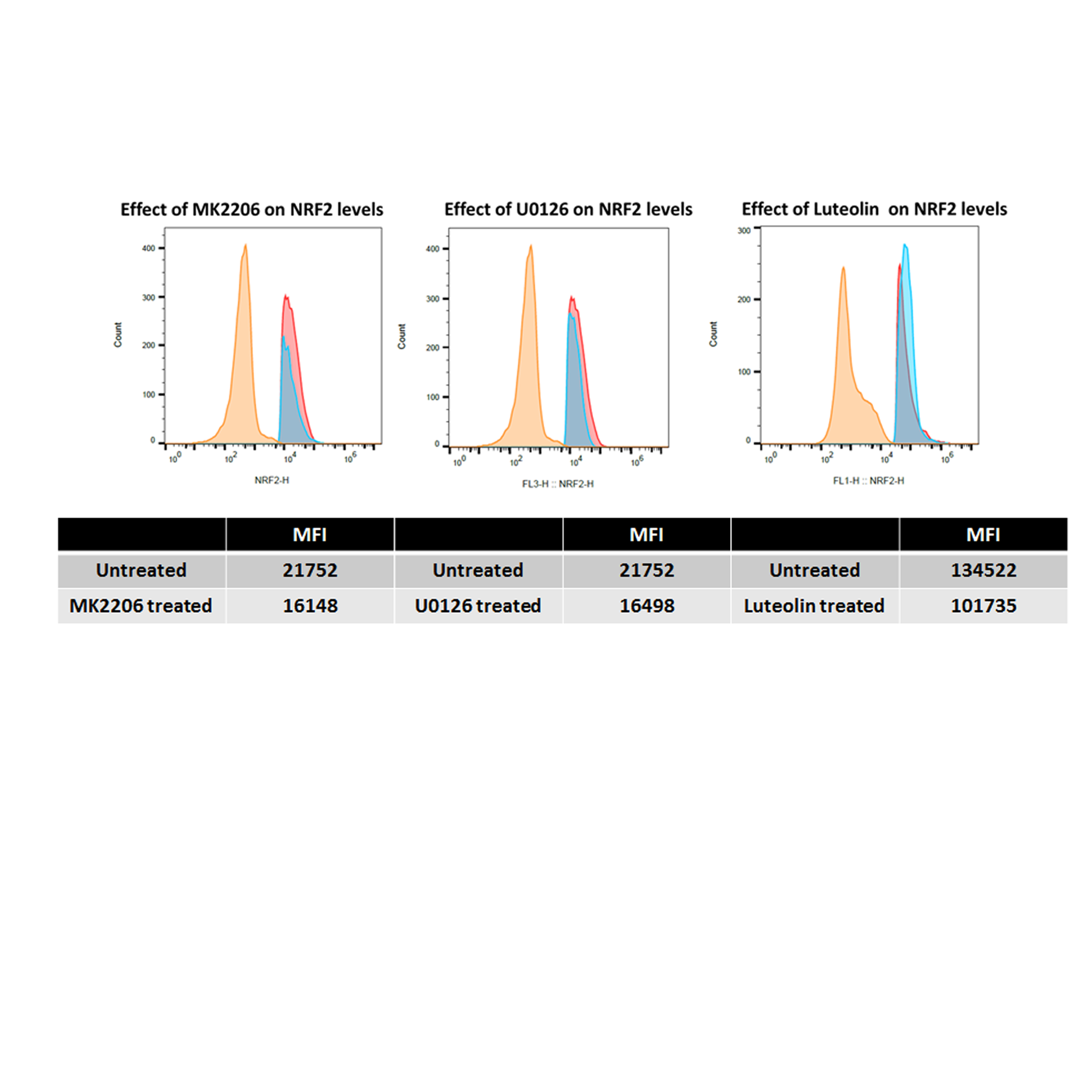

Supplement: S6 Fig — AML cell line THP1 was treated with (A) 10μM of MK2206 (B) 10μM of U0126 or (C) 40μM of Luteolin for 24hrs and expression of Nrf2 was measured by flow cytometry. (TIF) [file pone.0177227.s006.tif]

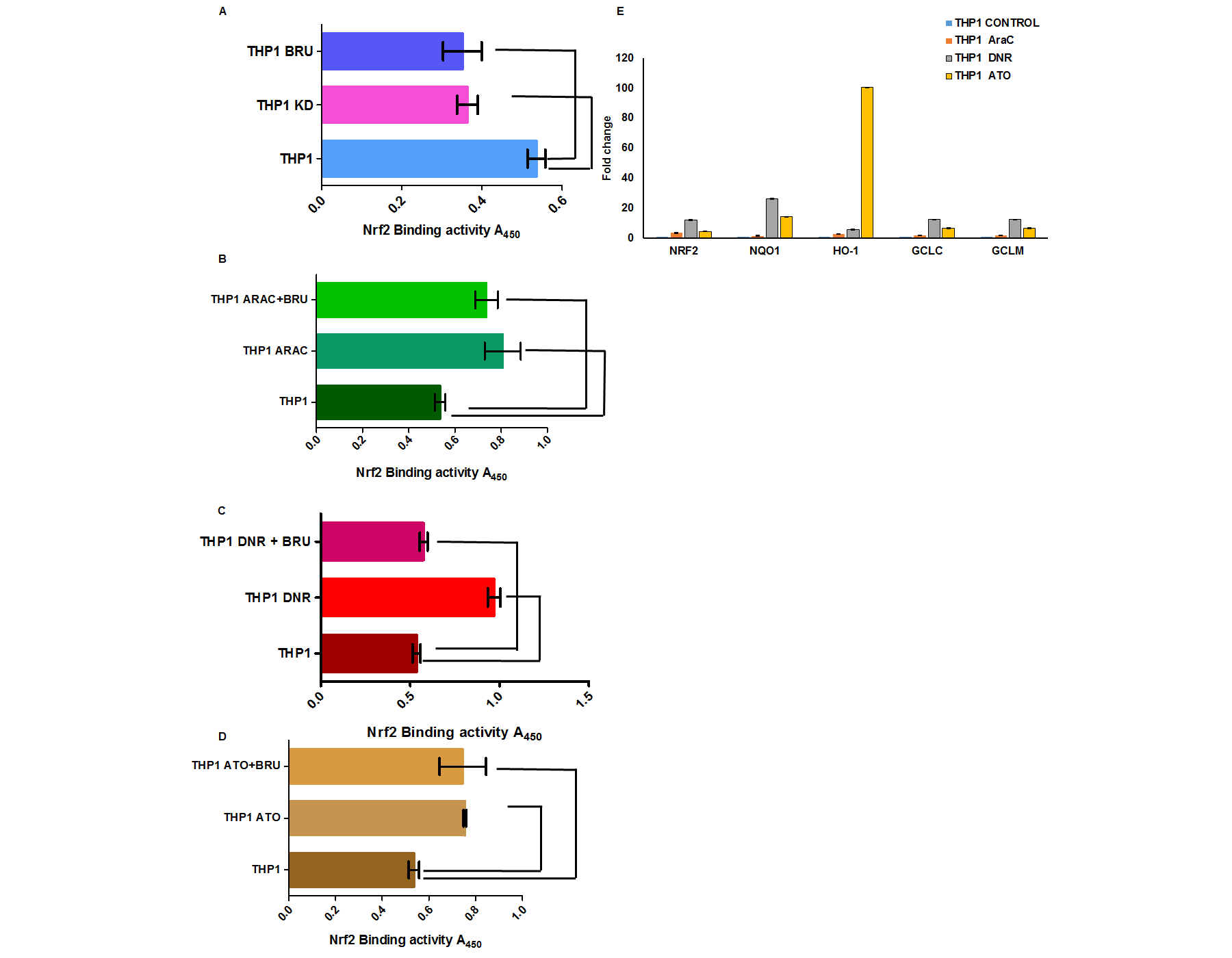

Supplement: S7 Fig — AML cell line THP1 was treated with and without 100nM of Brusatol for 6h. This was followed by treatment with Ara-C (5μM), Dnr (1μM) and ATO (6μM) for another 24h. Nuclear lysates were quantified and 6μg of protein was added per well. Nuclear lysates were also prepared from Nrf2 knock down THP1 cells. ARE binding activity was determined spectrophotometrically at 450nm. (A) Brusatol effectively reduced the ARE binding activity of Nrf2; similar effect was observed in Nrf2 knock down THP1 cells. Treatment of THP1 cells with chemotherapeutic agents Ara-C (B), Dnr (C) and ATO (D) increased the ARE activity as well as expression of downstream targets (E), while Brusatol co treatment reduced this activity. Brusatol reduced ARE activity moderately in Dnr and minimally in ATO and Ara-C treated cells. (TIF) [file pone.0177227.s007.tif]

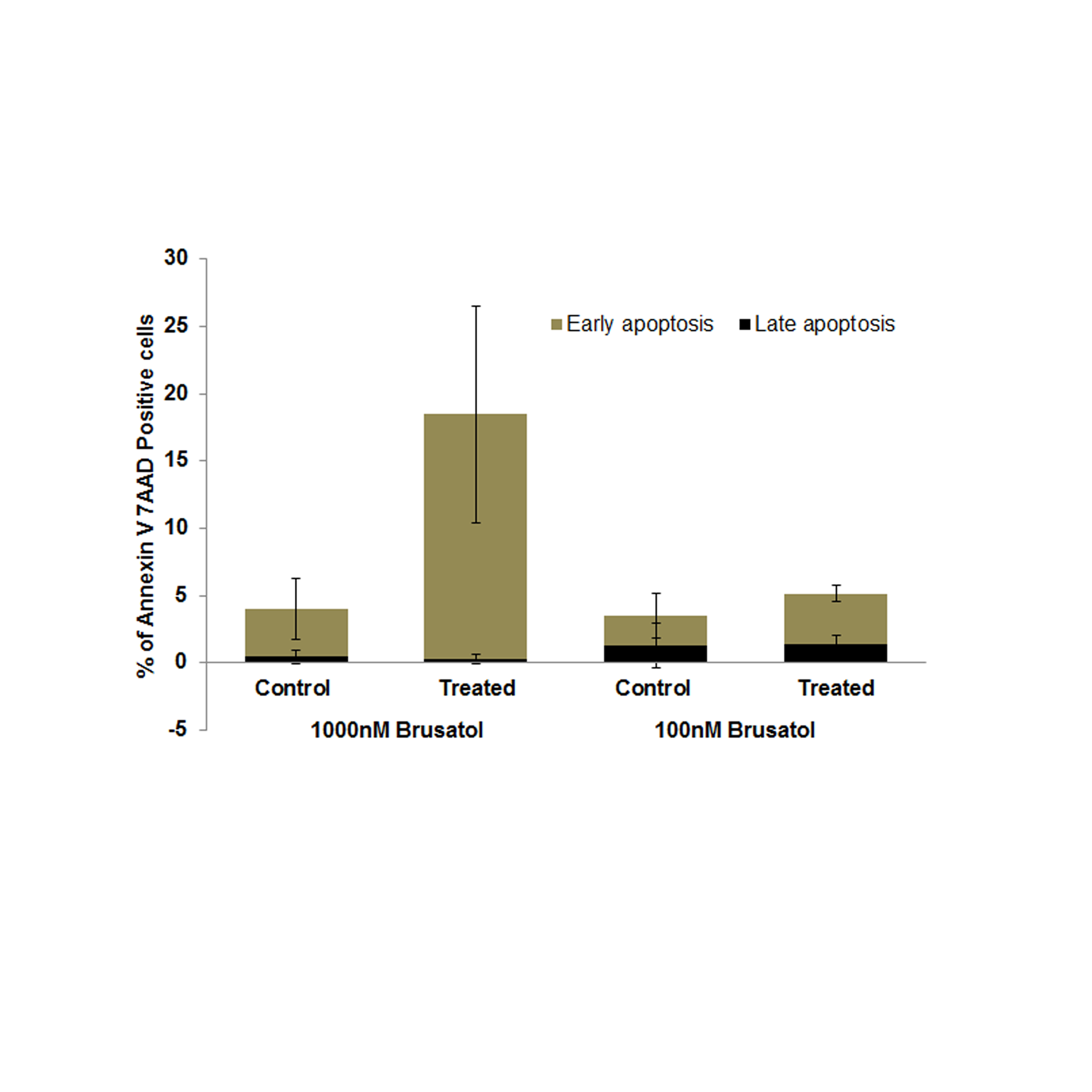

Supplement: S8 Fig — THP1 cells were treated with two different concentrations of Brusatol (100nM & 1000nM) and incubated for 6hrs. After incubation, cells were washed and stained with Annexin V 7AAD and the apoptosis was measured. Values represent mean ± SD of two independent experiments. (TIF) [file pone.0177227.s008.tif]

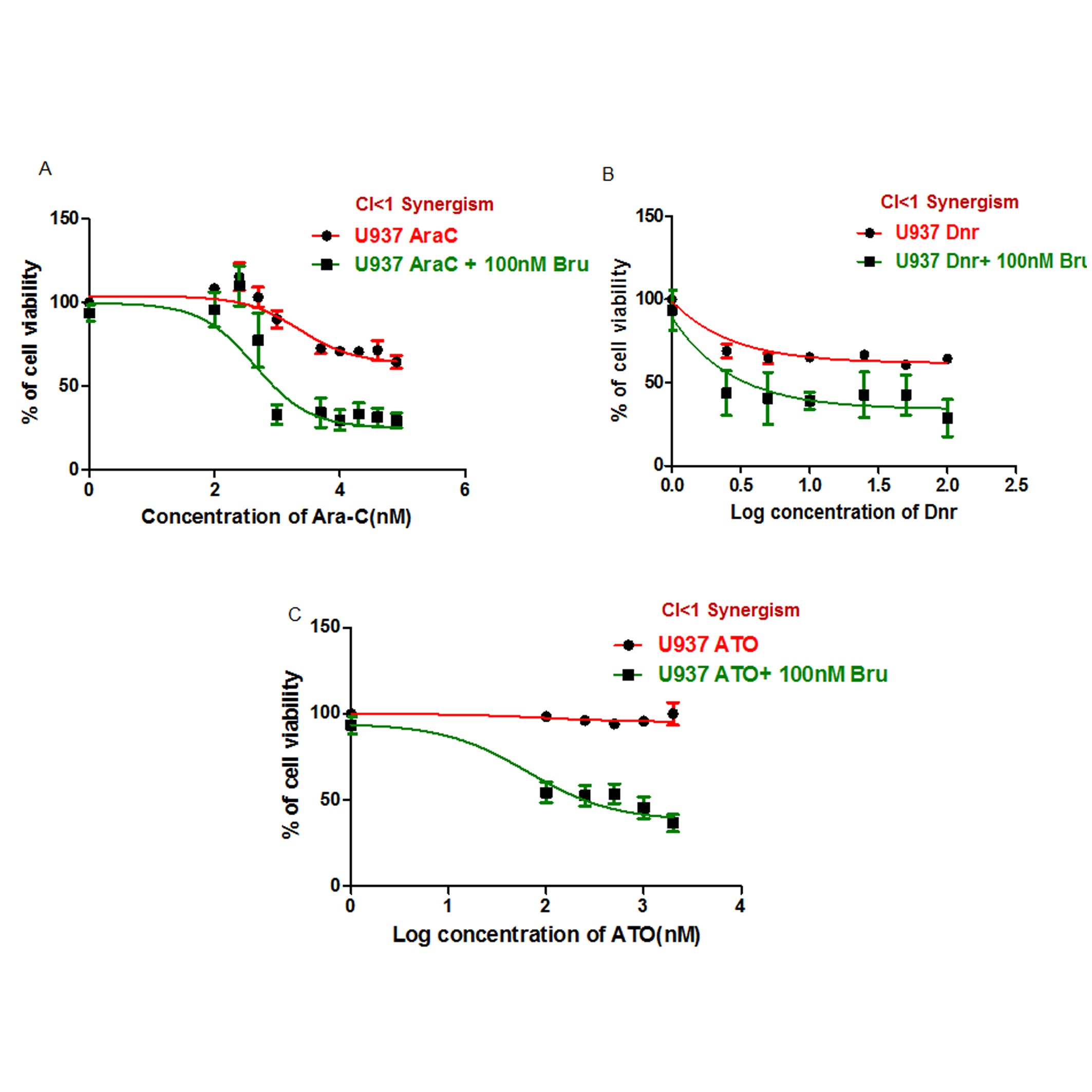

Supplement: S9 Fig — U937 cells were incubated with Nrf2 inhibitor Brusatol 100nM for 6hrs, followed by increasing concentration of (A) Ara-C, (B) Dnr and (C) ATO for 48hrs. In-vitro cytotoxicity was measured by MTT assay. (TIF) [file pone.0177227.s009.tif]

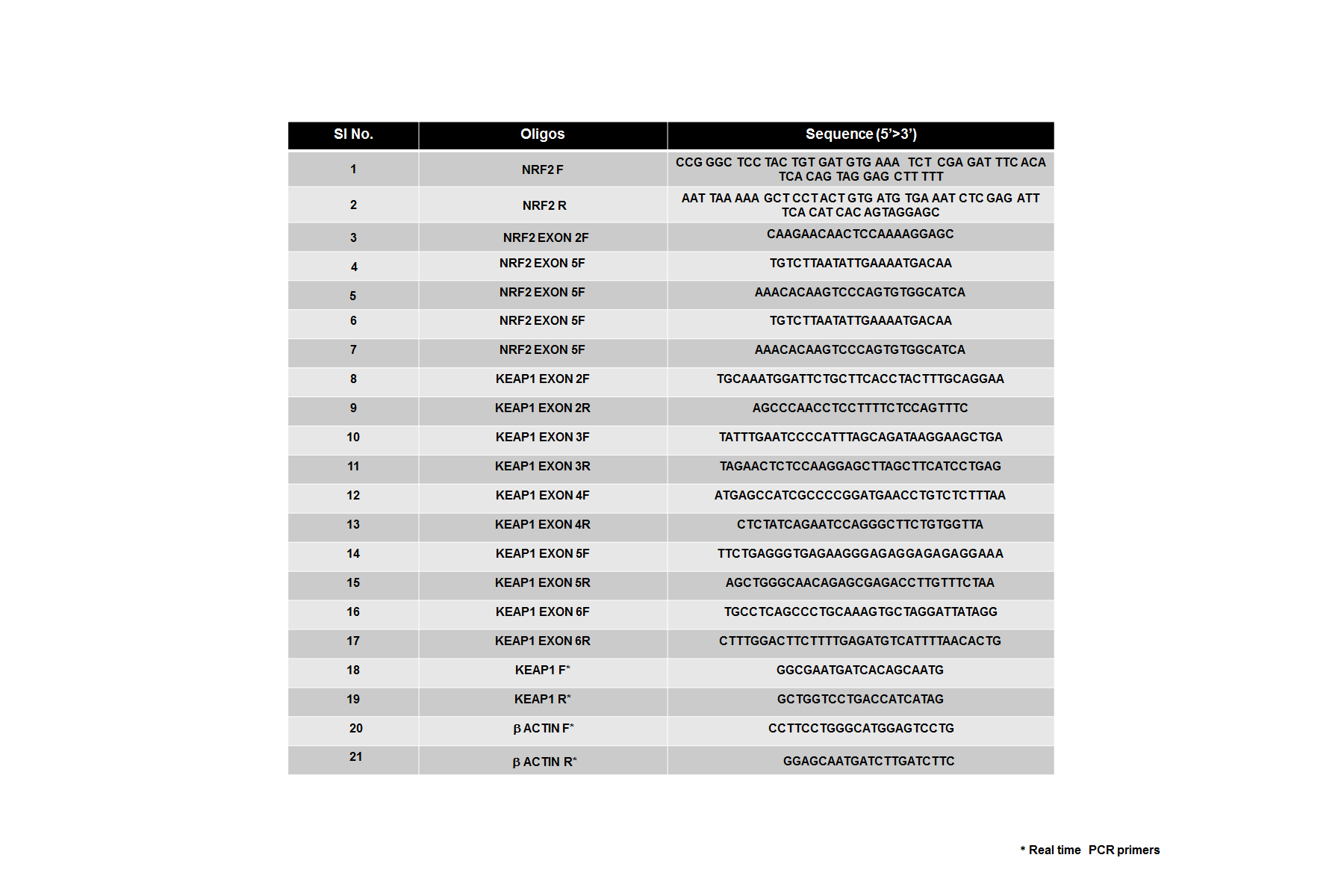

Supplement: S1 Table — (TIF) [file pone.0177227.s010.tif]

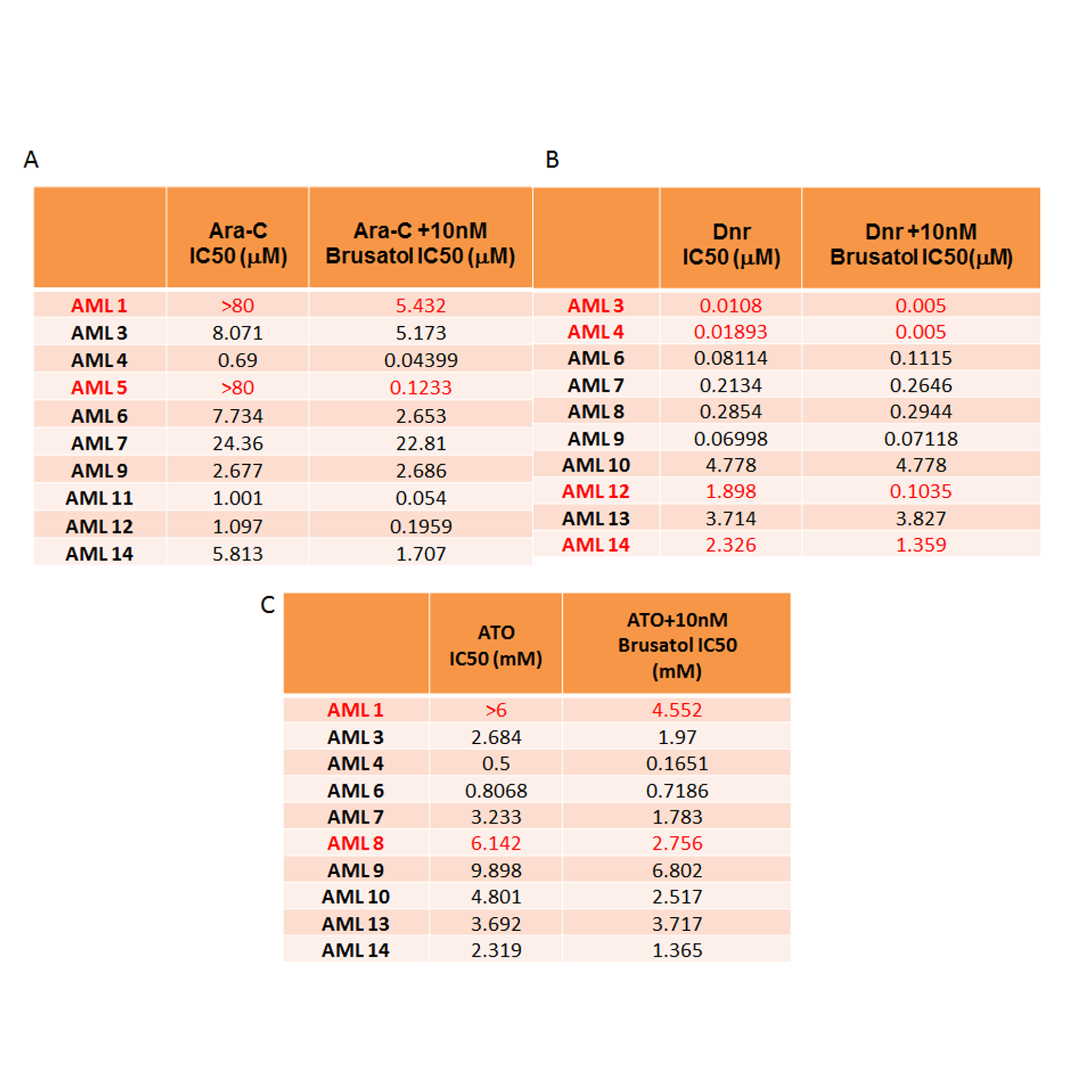

Supplement: S2 Table — Primary samples at diagnosis was subjected to pre-treatment with brusatol followed by increasing concentrations of (A) Ara-C (0.1–80μM), (B) Dnr (0.0025–1μM) and (C) ATO (0.1–6μM) for 48h. Ex -vivo cytotoxicity was measured by MTT assay. (TIF) [file pone.0177227.s011.tif]
